# Six novel steroids from culture of basidiomycete *Polyporus ellisii*

**DOI:** 10.1007/s13659-012-0058-4

**Published:** 2012-12-12

**Authors:** Shuang Wang, Ling Zhang, Liang-Yan Liu, Ze-Jun Dong, Zheng-Hui Li, Ji-Kai Liu

**Affiliations:** 1 State Key Laboratory of Phytochemistry and Plant Resources in West China, Kunming Institute of Botany, Chinese Academy of Sciences, Kunming, 650201 China; 2 University of Chinese Academy of Sciences, Beijing, 100049 China

**Keywords:** ergosterol, *Polyporus ellisii*, basidiomycete, cytotoxicity

## Abstract

Investigation of the culture of basidiomycete *Polyporus ellisii* led to the isolation of a novel compound 3*β*,9*α*,15*α*-trihydroxy-(22*E*,24*R*)-10(5→4)-*abeo*-ergosta-6,8(14),22-trien-5-one (**1**) with a new 5/7/6/5 ring system of ergosterol skeleton. In addition, five new steroids, 5*β*,6*β*-epoxy-3*β*,7*α*,9*α*-trihydroxy-(22*E*,24*R*)-ergosta-8(14),22-dien-15-one (**2**), 5*β*,6*β*-epoxy-3*β*,7*α*-dihydroxy-(22*E*,24*R*)-ergosta-8(14),22-dien-15-one (**3**), 5*α*,6*α*-epoxy-3*β*,9*α*,15*α*-trihydroxy-(22*E*,24*R*)-ergosta-8(14),22-dien-7-one (**4**), 15*α*-acetoxy-(22*E*,24*R*)-ergosta-4,6,8(14),22-tetraen-3-one (**5**), 15*β*-methoxy-(22*E*,24*R*)-ergosta-4,6,8(14),22-tetraen-3-one (**6**), along with four known ergosterols (**7–10**), were obtained. All structures were elucidated based on 1D and 2D NMR spectral data. New compounds were evaluated for cytotoxicity against five human cancer cell lines, only compound **4** was found to exhibit a favorable cytotoxicity profile toward all tested tumor cell lines.

**Electronic Supplementary Material:**

Supplementary material is available for this article at 10.1007/s13659-012-0058-4 and is accessible for authorized users.

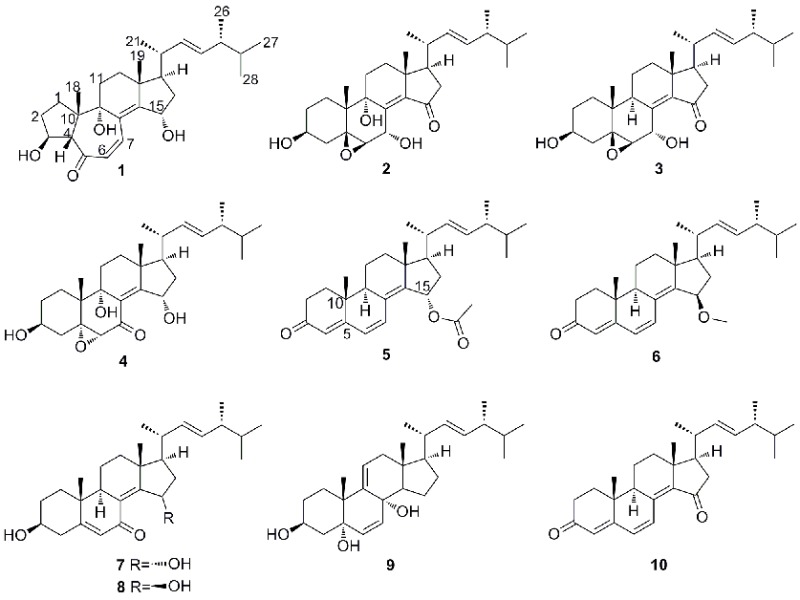

## Introduction

The fungus *Polyporus ellisii*, belonging to the family Polyporaceae, is widely distributed in Yunnan and Sichuan Provinces of China. We had previously isolated biologically active cerebrosides from the fruiting bodies of *P. ellisii*.[1–3] In this study, a novel compound 3*β*,9*α*,15*α*-trihydroxy-(22*E*,24*R*)-10(5→4)-*abeo*-ergosta-6,8(14),22-trien-5-one (**1**) with a new 5/7/6/5 ring system was obtained from the culture of *P. ellisii*. In addition, three new multi-oxygenbearing ergosterols, namely 5*β*,6*β*-epoxy-3*β*,7*α*,9*α*-trihydroxy-(22*E*,24*R*)-ergosta-8(14),22-dien-15-one (**2**), 5*β*,6*β*-epoxy-3*β*,7*α*-dihydroxy-(22*E*,24*R*)-ergosta-8(14),22-dien-15-one (**3**), 5*α*,6*α*-epoxy-3*β*,9*α*,15*α*-trihydroxy-(22*E*,24*R*)-ergosta-8(14),22-dien-7-one (**4**), and two derivatives of (22*E*,24*R*)-ergosta-4,6,8(14),22-tetraen-3-one, namely 15*α*-acetoxy-(22*E*, 24*R*)-ergosta-4,6,8(14),22-tetraen-3-one (**5**), 15*β*-methoxy-(22 *E*,24*R*)-ergosta-4,6,8(14),22-tetraen-3-one (**6**), were obtained. Four known ergosterols, 3*β*,15*α*-dihydroxy-(22*E*,24*R*)-ergosta-5,8(14),22-trien-7-one (**7**)[4], 3*β*,15*β*-dihydroxy-(22*E*,24*R*)-ergosta-5,8(14),22-trien-7-one (**8**)[4], (22*E*,24*R*)-ergosta-6,9,22-trien-3*β*,5*α*,8*α*-triol (**9**)[5], (22*E*,24*R*)-ergosta-4,6,8(14),22-tetraen-3,15-dione (gymnasterone, **10**)[6] were also isolated from the species. New compounds were evaluated for cytotoxicity against five human cancer cell lines. Compound **1** showed cytotoxic activity against four cell lines, and compound **4** exhibited a favorable cytotoxicity profile toward all tested tumor cell lines.

**Figure 1.** Structures of compounds **1**–**10**

## Results and Discussion

Compound **1**, obtained as a light yellow amorphous powder, had a molecular formula of $C_{28}H_{42}O_4$ on the basis of the positive HRESIMS, which showed a molecular ion peak at *m/z* 465.2980 (calcd for $C_{28}H_{42}O_4Na$), and accounted for 8 degrees of unsaturation. The presence of a conjugated system was confirmed by UV spectrum which showed maximum absorption at 292 nm. The IR spectrum indicated the presence

*To whom correspondence should be addressed. E-mail: jkliu@mail.kib.ac.cn of hydroxy (3432 $cm^{-1}$), carbonyl (1738 $cm^{-1}$) and double-bond (1664 $cm^{-1}$) groups. The $^{13}C$ NMR and DEPT spectra (Table 2) exhibited 28 signals: 6 methyls, 5 methylenes, 4 $sp^2$ and 7 $sp^3$ methines, and 3 $sp^2$ and 3 $sp^3$ quaternary carbons. The four degrees of unsaturation for the formula were attributed to three sets of olefinic groups at $\delta_C$ 123.5, 145.9, 134.8, 165.4, 133.8, 133.9, and an unsaturated ketone carbonyl carbon at $\delta_C$ 203.8. The remaining degrees of unsaturation were thus attributed to the presence of four rings. The $^1H$ NMR spectrum (Table 1) also showed six methyl protons at highfield ($\delta_H$ 1.23 s, 1.07 s, 1.06 d, 0.92 d, 0.86 d, 0.82 d). Based on the above analysis, compound **1** was suggested to be an ergosterol. Rings C and D of **1** were elucidated as the same as those of compound **7**,[4] and confirmed by HMBC and COSY correlations. Compared to the other ergosterols isolated from the same fungus, the differences were the presence of changed rings A and B. The unsaturated ketone carbonyl carbon at $\delta_C$ 203.8 was located at C-5, confirmed by HMBC correlations between H-7, H-4, H-3/C-5. The olefinic group at $\delta_C$ 123.5, 145.9 ($\delta_H$ 5.89 d, 7.52 d) was attributed to C-6 and C-7. Since the two olefinic protons coupled each other in $^1H$-$^1H$ COSY spectrum and there existed HMBC correlations from H-6 to C-4, C-7, C-8 and from H-7 to C-5, C-6, C-8, C-9. The HMBC correlations mentioned above implied the existence of an unusual seven-membered ring B. A combination of COSY and HSQC showed the connectivities C-4–C-3–C-2–C-1 for ring A. The existence of seven-membered ring B and five-membered ring A suggested the two rings were connected by single bond between C-4 and C-10, which was confirmed by HMBC correlations between H-4/C-18, Me-18/C-1, C-9, C-10, H-1/C-9 and H-3/C-5. Thus, the skeleton of **1** was established as an ergosterol with a rearranged 10(5→4)-*abeo* skeleton.

The ROESY correlations of **1** (Figure 2) between H-15/Me-19 and H-4/Me-18 demonstrated that the stereochemistry of H-15 and H-4 were *β*-oriented, respectively. No any correlations in ROESY (in DMSO-$d_6$) between 9-OH and Me-18 or Me-19 were observed, which suggested the existence of 9*α*-OH. The ROESY (in DMSO-$d_6$) correlations between 9-OH and H-3 indicated the existence of 3*β*-OH. The stereochemistry of the side chain was established by the comparison with the known compounds (**7**–**10**)[4–6] and literature data of similar compounds[7,8]. Thus, **1** was elucidated as 3*β*,9*α*,15*α*-trihydroxy-(22*E*,24*R*)-10(5→4)-*abeo*-ergosta-6,8(14),22-trien-5-one.

**Figure 2.** The main HMBC, COSY and ROESY correlations of **1**–**6**

Compound **2** was found to possess a molecular formula of $C_{28}H_{42}O_5$ with 8 degrees of unsaturation, as evidenced by positive HRESIMS at *m/z* 481.2929 (calcd for $C_{28}H_{42}O_5Na$), in combination with $^1H$ NMR, $^{13}C$ NMR and DEPT spectral data.

**Table 1.** $^1H$ **NMR data of compounds 1–6 in** $CDCl_3$

| pos. | **1** | **2** | **2** (in DMSO-$d_6$) | **3** | **4** | **5** | **6** |
|---|---|---|---|---|---|---|---|
| 1 | 1.70 m; 1.89 m | 1.66 m; 1.84 m | 1.98 m; 1.41 m | 1.50 m; 1.68 m | 1.85 m; 2.32 m | 1.84 m; 2.03 m | 1.83 m; 2.03 m |
| 2 | 1.55 m; 1.82 m | 2.05 m; 1.59 m | 1.83 m; 1.41 m | 1.50 m; 1.97 m | 1.61 m; 2.08 m | 2.49 m; 2.54 m | 2.48 m; 2.54 m |
| 3 | 4.26 m | 3.96 m | 3.57 m | 3.95 br. s | 3.96 m | | |
| 4 | 2.65 d (9.6) | 1.43 m; 2.20 m | 1.18 m; 2.04 m | 2.06 m; 2.21 m | 1.47 m; 2.30 m | 5.77 s | 5.77 s |
| 6 | 5.89 d (10.5) | 3.35 d (3.2) | 3.24 d (3.7) | 3.17 d (3.0) | 3.37 s | 6.10 d (9.6) | 6.12 d (9.6) |
| 7 | 7.52 d (10.5) | 5.39 dd (5.3, 3.2) | 5.50 dd (3.7, 4.7) | 5.13 d (3.0) | | 6.61 d (9.6) | 6.75 d (9.6) |
| 9 | | | | 2.79 t (8.4) | | 2.17 m | 2.17 m |
| 11 | 1.43 m; 1.86 m | 2.22 m; 1.59 m | 1.39 m; 1.71 m | 1.51 m; 1.56 m | 1.70 m; 1.92 m | 1.72 m; 1.64 m | 1.70 m; 1.63 m |
| 12 | 1.82 m; 2.09 m | 1.93 m; 1.84 m | 1.56 m; 1.79 m | 1.40 m; 2.10 m | 1.92 m; 1.83 m | 1.26 m; 2.54 m | 2.23 m; 2.06 m |
| 15 | 4.89 d (5.8) | | | | 4.92 m | 5.78 d (7.3) | 4.33 m |
| 16 | 1.70 m; 2.24 m | 2.38 dd (19.0, 7.5); 2.19 m | 2.16 dd (19.2, 8.0); 2.02 m | 2.19 m; 2.29 m | 1.82 m; 1.89 m | 2.43 m; 1.42 m | 1.24 m; 1.49 m |
| 17 | 1.70 m | 1.67 m | 1.49 m | 1.54 m | 1.81 m | 1.22 m | 1.16 m |
| 18 | 1.23 s | 1.01 s | 0.87 s | 0.92 s | 1.08 s | 1.03 s | 1.05 s |
| 19 | 1.07 s | 1.02 s | 0.89 s | 1.02 s | 0.93 s | 1.10 s | 1.09 s |
| 20 | 1.43 m | 2.22 m | 2.18 m | 1.47 m | 2.12 m | 2.18 m | 2.19 m |
| 21 | 1.06 d (6.8) | 1.09 d (6.8) | 1.04 d (6.8) | 1.08 d (6.8) | 1.05 d (6.8) | 1.06 d (6.8) | 1.05 d (6.8) |
| 22 | 5.23 m | 5.16 dd (15.1, 8.4) | 5.19 dd (15.2, 8.3) | 5.15 dd (15.1, 8.3) | 5.22 m | 5.16 dd (15.2, 8.0) | 5.18 dd (15.2, 8.8) |
| 23 | 5.23 m | 5.27 dd (15.1, 8.4) | 5.28 dd (15.2, 8.3) | 5.27 dd (15.1, 8.3) | 5.22 m | 5.25 dd (15.2, 8.0) | 5.28 dd (15.2, 8.8) |
| 24 | 1.87 m | 1.86 m | 1.86 m | 1.85 m | 1.85 m | 1.85 m | 1.87 m |
| 25 | 1.47 m | 1.47 m | 1.46 m | 1.27 m | 1.46 m | 1.46 m | 1.48 m |
| 26 | 0.92 d (6.8) | 0.91 d (6.8) | 0.88 d (6.8) | 0.91 d (6.8) | 0.91 d (6.8) | 0.92 d (6.8) | 0.93 d (6.8) |
| 27 | 0.82 d (6.8) | 0.81 d (6.8) | 0.79 d (6.8) | 0.81 d (6.8) | 0.81 d (6.8) | 0.81 d (6.8) | 0.83 d (6.8) |
| 28 | 0.84 d (6.8) | 0.83 d (6.8) | 0.80 d (6.8) | 0.83 d (6.8) | 0.83 d (6.8) | 0.83 d (6.8) | 0.84 d (6.8) |
| 3-OH | | | 4.81 d (4.5) | | | | |
| 7-OH | | 4.16 d (5.3) | 4.92 d (5.3) | | | | |
| 9-OH | | 5.28 br. s | 4.72 d (2.3) | | | | |
| 1'-$CH_3$ | | | | | | | 3.26 s |
| 2'-$CH_3$ | | | | | | 2.03 s | |

**Table 2.** $^{13}$**C NMR data of compounds 1–10 in CDCl$_3$**

| pos. | 1 | 2 | 2 (in DMSO-$d_6$) | 3 | 4 | 5 | 6 | 7 | 8 | 9 | 10 |
|---|---|---|---|---|---|---|---|---|---|---|---|
| 1 | 37.2 | 26.8 | 25.8 | 32.2 | 26.5 | 33.9 | 34.0 | 35.2 | 36.2 | 32.5 | 33.9 |
| 2 | 27.3 | 30.4 | 30.4 | 30.8 | 30.5 | 34.0 | 34.0 | 31.3 | 31.8 | 30.5 | 33.9 |
| 3 | 67.0 | 68.0 | 66.7 | 68.3 | 68.2 | 199.5 | 199.6 | 70.0 | 70.6 | 66.3 | 199.0 |
| 4 | 58.0 | 39.8 | 40.0 | 39.1 | 39.3 | 124.0 | 123.6 | 41.6 | 42.5 | 36.0 | 125.1 |
| 5 | 203.8 | 67.4 | 68.1 | 64.4 | 70.6 | 163.3 | 163.6 | 168.6 | 173.7 | 82.7 | 161.9 |
| 6 | 123.5 | 60.9 | 60.8 | 60.1 | 62.3 | 126.9 | 126.3 | 127.6 | 127.4 | 135.4 | 131.3 |
| 7 | 145.9 | 62.9 | 59.7 | 62.9 | 196.7 | 132.3 | 132.9 | 190.2 | 193.2 | 130.7 | 131.5 |
| 8 | 134.8 | 145.2 | 142.3 | 149.3 | 130.0 | 130.4 | 130.0 | 126.2 | 130.1 | 78.3 | 139.0 |
| 9 | 81.3 | 75.2 | 74.6 | 41.6 | 74.4 | 44.4 | 44.8 | 47.3 | 48.2 | 142.5 | 45.2 |
| 10 | 40.5 | 37.1 | 37.1 | 39.1 | 38.6 | 36.8 | 36.7 | 38.4 | 41.2 | 37.9 | 37.6 |
| 11 | 39.4 | 26.1 | 26.5 | 18.9 | 26.5 | 18.9 | 18.9 | 19.3 | 20.2 | 119.7 | 18.7 |
| 12 | 35.3 | 32.5 | 32.5 | 36.0 | 32.1 | 35.7 | 36.1 | 35.4 | 36.6 | 41.1 | 35.6 |
| 13 | 50.5 | 43.0 | 42.6 | 42.1 | 47.8 | 43.2 | 43.0 | 44.8 | 46.9 | 43.4 | 42.6 |
| 14 | 165.4 | 144.1 | 143.9 | 143.2 | 180.0 | 150.7 | 152.3 | 168.0 | 170.0 | 48.1 | 145.3 |
| 15 | 71.9 | 211.0 | 207.5 | 210.0 | 71.1 | 70.7 | 77.3 | 70.6 | 71.2 | 28.6 | 206.6 |
| 16 | 36.3 | 43.6 | 42.4 | 43.4 | 36.5 | 36.8 | 36.2 | 35.8 | 37.6 | 20.8 | 42.5 |
| 17 | 52.6 | 50.0 | 49.1 | 50.3 | 53.0 | 53.6 | 53.3 | 51.3 | 54.7 | 55.8 | 51.0 |
| 18 | 27.0 | 20.6 | 20.0 | 17.4 | 20.5 | 16.7 | 16.8 | 18.9 | 19.4 | 25.4 | 17.0 |
| 19 | 16.3 | 18.3 | 17.2 | 20.0 | 18.8 | 19.4 | 19.5 | 19.7 | 19.8 | 12.9 | 20.0 |
| 20 | 35.9 | 39.0 | 38.4 | 39.5 | 38.6 | 39.3 | 39.5 | 38.4 | 39.9 | 39.8 | 39.5 |
| 21 | 23.4 | 21.3 | 21.1 | 21.4 | 21.4 | 21.2 | 21.2 | 21.5 | 22.0 | 20.7 | 21.3 |
| 22 | 133.9 | 133.8 | 134.4 | 134.0 | 133.0 | 134.4 | 134.6 | 134.6 | 136.4 | 135.1 | 133.8 |
| 23 | 133.8 | 133.8 | 132.5 | 133.6 | 134.4 | 133.1 | 133.0 | 133.0 | 134.1 | 132.4 | 133.8 |
| 24 | 43.0 | 42.9 | 42.1 | 42.8 | 42.8 | 42.9 | 42.8 | 43.0 | 44.4 | 42.7 | 42.9 |
| 25 | 33.0 | 33.0 | 32.7 | 33.0 | 33.0 | 33.0 | 33.0 | 33.0 | 34.4 | 33.0 | 33.0 |
| 26 | 17.6 | 17.7 | 17.5 | 17.6 | 17.6 | 17.7 | 17.6 | 17.6 | 18.2 | 17.5 | 17.7 |
| 27 | 19.7 | 19.6 | 19.5 | 19.7 | 19.7 | 19.7 | 19.6 | 19.8 | 20.2 | 19.6 | 20.0 |
| 28 | 20.0 | 20.0 | 19.9 | 20.0 | 20.0 | 20.0 | 20.0 | 20.0 | 20.5 | 19.9 | 20.0 |
| 1' | | | | | | 170.6 | 56.1 | | | | |
| 2' | | | | | | 21.4 | | | | | |

The $^1$H NMR spectrum also exhibited characteristic ergosterol signals for six methyls at $\delta_H$ 1.02 s, 1.01 s, 1.09 d, 0.91 d, 0.83 d, and 0.81 d. The $^{13}$C NMR spectrum showed a carbonyl carbon at $\delta_C$ 211.0 and five oxygen-bearing carbons at $\delta_C$ 75.2, 68.0, 67.4, 62.9 and 60.9, implying the existence of an epoxy and three hydroxyl groups. The HMBC (in DMSO-$d_6$) correlations from the proton at $\delta_H$ 4.72 to C-9 ($\delta_C$ 74.6) and from the proton at $\delta_H$ 4.81 (d) to C-3 ($\delta_C$ 66.7) demonstrated the presence of hydroxyl group at C-9 and C-3, respectively. The HMBC correlations from the proton of hydroxyl group at $\delta_H$ 4.16 to C-6 ($\delta_C$ 60.9) and C-7 ($\delta_C$ 62.9), with the signals of H-6 ($\delta_H$ 3.35) and H-7 ($\delta_H$ 5.39) in $^1$H NMR spectrum appear as doublet (3.2) and double doublet (5.3, 3.2), respectively, suggested the presence of 5,6-epoxy. The stereochemistry of 9-OH was $\alpha$-oriented due to the skeleton of B/C *trans*.[9,10] The ROESY correlations (in DMSO-$d_6$) between H-7 ($\delta_H$ 5.50)/Me-18 ($\delta_H$ 0.87), 7-OH ($\delta_H$ 4.92)/9-OH ($\delta_H$ 4.72), 7-OH ($\delta_H$ 4.92)/H-6 ($\delta_H$ 3.24) and between H-6 ($\delta_H$ 3.24)/ H-4$\alpha$ ($\delta_H$ 1.18), suggested the presence of 7$\alpha$-OH and 5$\beta$,6$\beta$-epoxy. The stereochemistry of H-3 was determined to be $\alpha$-oriented due to its downfield shifted broad multiplet at $\delta_H$ 3.96 ($W_{1/2}$ = 20.0 Hz)[10,11]. The stereochemistry of the side chain of **2** was established by the comparison with the known compounds (**7**–**10**)[4–6] and literature data of similar compounds.[7,8] Thus, **2** was assigned as 5$\beta$,6$\beta$-epoxy-3$\beta$,7$\alpha$,9$\alpha$-trihydroxy-(22*E*,24*R*)-ergosta-8(14),22-dien-15-one.

The NMR data of compounds **3** and **4** were similar to those of **2**. The only difference between **3** and **2** appears to be the group at C-9. The hydroxyl at C-9 of **2** was replaced by a proton in compound **3**, which was confirmed by HMBC correlations from H-9 to C-10, C-11, C-14, and C-8. The ROESY correlations between H-7 ($\delta_H$ 5.13)/Me-18 ($\delta_H$ 0.92) and H-6 ($\delta_H$ 3.17)/H-9 ($\delta_H$ 2.79) demonstrated the existence of 7$\alpha$-OH and 5$\beta$,6$\beta$-epoxy. Thus, the structure of **3** was identified as 5$\beta$,6$\beta$-epoxy-3$\beta$,7$\alpha$-dihydroxy-(22*E*,24*R*)-ergosta-8(14),22-dien-15-one.

The differences between **4** and **2** appears to be the groups at C-7 and C-15 and the stereochemistry of 5,6-epoxy. The HMBC correlations of **4** from H-6 ($\delta_H$ 3.37) to C-7 ($\delta_C$ 196.7) and from H-15 ($\delta_H$ 4.92) to C-13 ($\delta_C$ 47.8), C-16 ($\delta_C$ 36.5) and C-17 ($\delta_C$ 53.0), proved that the groups at C-7 and C-15 were carbonyl and hydroxyl, respectively. The ROESY correlations between H-15 ($\delta_H$ 4.92)/Me-19 ($\delta_H$ 0.93), H-6 ($\delta_H$ 3.37)/Me-18 ($\delta_H$ 1.08), H-6 ($\delta_H$ 3.37)/H-4$\beta$ ($\delta_H$ 2.29) and Me-18 ($\delta_H$ 1.08)/H-4$\beta$ ($\delta_H$ 2.29), demonstrated the existence of 15$\alpha$-OH and 5$\alpha$,6$\alpha$-epoxy. Thus, **4** was characterized as 5$\alpha$,6$\alpha$-epoxy-3$\beta$,9$\alpha$,15$\alpha$-trihydroxy-(22*E*,24*R*)-ergosta-8(14),22-dien-7-one.

Compound **5** was obtained as a light yellow amorphous powder. The molecular formula was established to be $C_{30}H_{42}O_3$ on the basis of the positive HRESIMS at *m/z* 451.3212 (calcd for $C_{30}H_{42}O_3H$, 451.3221). The UV spectrum showed the maximum absorption at 333 nm, indicating the existence of a long conjugation system. As shown in Tables 1 and 2, the NMR data showed six characteristic ergosterol methyls. Actually, the data were similar to those of (22*E*,24*R*)-ergosta-4,6,8(14),22-tetraen-3-one,[12] with the only difference at the group of C-15. The HMBC correlations from H-15 ($\delta_H$ 5.78) to C-1' ($\delta_C$ 170.6) and from Me-2' ($\delta_H$ 2.03) to C-1' ($\delta_C$ 170.6) demonstrated the existence of acetoxy group at C-15 in **5**. The ROESY correlations between H-15 ($\delta_H$ 5.78)/Me-19 ($\delta_H$ 1.10) indicated the acetoxy group at C-15 was $\alpha$-oriented. Based on the above evidence, **5** was elucidated as 15$\alpha$-acetoxy-(22*E*,24*R*)-ergosta-4,6,8(14),22-tetraen-3-one. The NMR data of **6** were similar with those of **5**, expect that acetoxy group at C-15 in **5** was replaced by methoxy group. The ROESY correlations between H-15 ($\delta_H$ 4.33)/H-17 ($\delta_H$ 1.16) showed that the methoxy group at C-15 was $\beta$-oriented. Thus, **6** was deduced to be 15$\beta$-methoxy-(22*E*,24*R*)-ergosta-4,6,8(14),22-tetraen-3-one.

The isolated new sterols (**1**–**6**) were evaluated for cytotoxicity against five human tumor cell lines using MTT method (Table 3). **4** showed significant cytotoxicity to all tested tumor cell lines.

Comparison of the physicochemical properties with the reported data allowed to identify compounds **7**–**10**, isolated from the same fungus, as 3*β*,15*α*-dihydroxy-(22*E*,24*R*)-ergosta-5,8(14),22-trien-7-one (**7**)[4], 3*β*,15*β*-dihydroxy-(22*E*,24*R*)-ergosta-5,8(14),22-trien-7-one (**8**)[4], (22*E*,24*R*)-ergosta-6,9,22-trien-3*β*,5*α*,8*α*-triol (**9**)[5], (22*E*,24*R*)-ergosta-4,6,8(14),22-tetraen-3,15-dione (gymnasterone, **10**)[6], respectively.

**Table 3. Cytotoxicity data of compounds 1–6[a]**

| compound | HL-60 | SMMC-7721 | A-549 | MCF-7 | SW480 |
|---|---|---|---|---|---|
| **1** | 17.1 | 21.3 | > 40 | 23.3 | 16.3 |
| **2** | 18.8 | > 40 | > 40 | > 40 | > 40 |
| **3** | 32.1 | > 40 | > 40 | > 40 | > 40 |
| **4** | 1.5 | 3.9 | 2.7 | 3.1 | 2.9 |
| **5** | 22.8 | > 40 | > 40 | > 40 | > 40 |
| **6** | 17.8 | > 40 | > 40 | > 40 | > 40 |
| DDP | 1.8 | 8.9 | 11.7 | 15.9 | 16.7 |
| Taxol | < 0.008 | < 0.008 | < 0.008 | < 0.008 | < 0.008 |

[a]Data were expressed in $IC_{50}$ values (*μ*M). DDP and taxol were used as positive controls.

## Experimental Section

**General Experimental Procedures.** Optical rotations were measured on a Jasco-P-1020 polarimeter. IR spectra were obtained by using a Bruker Tensor 27 FT-IR spectrometer with KBr pellets. NMR spectra were acquired with instruments of Avance III 600, Bruker DRX-500 or Bruker AV 400. ESIMS and HSESIMS were measured on Bruker HCT/Esquire and API QSTAR Pulsar, respectively. Silica gel (200–300 mesh and 80–100 mesh, Qingdao Marine Chemical Inc., China) and Sephadex LH-20 (Amersham Biosciences, Sweden) were used for column chromatography. Fractions were monitored by TLC and spots were visualized by heating silica gel plates immersed in vanillin-$H_2SO_4$ in EtOH, in combination with Agilent 1200 series HPLC system (Eclipse XDB-C18 column, 5 *μ*m, 4.6 × 150 mm). Preparative HPLC was performed on an Agilent 1100 series with a Zorbax SB-C18 (5 mm, 9.4 × 150 mm) column. Preparative MPLC was performed on Büchi apparatus equipped with Büchi fraction collector C-660, Büchi pump module C-605 and manager C-615.

**Fungal Material and Cultivation Conditions.** Fruiting bodies of *P. ellisii* were collected at Jingdong, Yunnan Province, China in 2003 and identified by Prof. Zhu-Liang Yang (Kunming Institute of Botany). The voucher specimen (NO.CGBWSHF00118) was deposited at herbarium of Kunming Institute of Botany. Culture medium: glucose (5%), pork peptone (0.15%), yeast (0.5%), $KH_2PO_4$ (0.05%), $MgSO_4$ (0.05%), The initial pH was adjusted to 6.0, the fermentation was first carried out on an erlenmeyer flask for six days till the mycelium biomass reached to the maximum. Later it was transferred to a fermentation tank (100 L) at 24 °C and 250 rpm for twenty days, ventilation was set to 1.0 vvm (vvm: air volume/culture volume/min).

**Extraction and Isolation.** The culture broth (80 L) was extracted four times with EtOAc. The organic layer was evaporated in vacuo to give a crude extract (71 g), which was applied on silica gel column chromatography (200–300 mesh) eluted with a petroleum ether-acetone gradient system to afford fractions A–G. Fraction C, eluted with petroleum ether-acetone (8/1), was separated by Sephadex LH-20 ($CHCl_3$-MeOH, 1/1) column chromatography and then applied to preparative MPLC with a reversed-phased C-18 column (MeOH-$H_2O$, 50%–100%) to give subfractions C1 and C2. Both subfractions was further purified by preparative HPLC (MeCN-$H_2O$, 80%–100%, 10 mL/min) to yield **5** (1.9 mg) from C1, **6** (2.4 mg) from C1, **10** (4.8 mg) from C1, and **9** (5.0 mg) from C2. Fraction D, eluted with petroleum ether-acetone (5/1), was further isolated and purified by silica gel ($CHCl_3$-$Me_2CO$, 10/1), Sephadex LH-20 ($CHCl_3$-MeOH, 1/1) and reversed-phased MPLC (MeOH-$H_2O$, 50%–100%) to get subfractions D1 and D2. Both subfractions were then purified by preparative HPLC (MeCN-$H_2O$, 79%–85%, 10 mL/min) to obtain **1** (4.0 mg) from D1, **7** (5.0 mg) from D2, and **8** (5.0 mg) from D2. Fraction E, eluted with petroleum ether-acetone (1/1), was further separated on silica gel ($CHCl_3$-$Me_2CO$, 5/1), reversed-phased MPLC (MeOH-$H_2O$, 30%–60%) and HPLC (MeCN-$H_2O$, 0%–20%, 10 mL/min) to give **2** (4.3 mg), **3** (4.5 mg), and **4** (4.5 mg).

**3*β*,9*α*,15*α*-trihydroxy-(22*E*,24*R*)-10(5→4)-*abeo*-ergosta-6,8(14),22-trien-5-one (1):** light yellow amorphous powder, $[\alpha]^{17.3}_{D}$ – 96.8 (*c* 0.05 MeOH); IR (KBr) $\nu_{max}$ 3432, 2956, 2925, 2871, 1728, 1664, 1461 $cm^{-1}$; UV (MeOH) $\lambda_{max}$ (log ε) 292 nm (3.29), 200 nm (3.16); $^1H$ NMR data (see Table 1); $^{13}C$ NMR data (see Table 2); ESIMS (pos.) *m/z* 465 $[M + Na]^+$; HRESIMS (pos.) *m/z* 465.2980 (calcd for $C_{28}H_{42}O_4Na$, 465.2980).

**5*β*,6*β*-epoxy-3*β*,7*α*,9*α*-trihydroxy-(22*E*,24*R*)-ergosta-8(14),22-dien-15-one (2):** amorphous powder, $[\alpha]^{20.6}_{D}$ – 7.1 (*c* 0.10 MeOH); IR (KBr) $\nu_{max}$ 3425, 2958, 2871, 1706, 1626, 1461, 1382, 1196 $cm^{-1}$; UV (MeOH) $\lambda_{max}$ (logε) 251 nm (3.27), 221 nm (3.44), 200 nm (3.35); $^1H$ NMR data (see Table 1); $^{13}C$ NMR data (see Table 2); ESIMS (pos.) *m/z* 481 $[M + Na]^+$; HRESIMS (pos.) *m/z* 481.2929 (calcd for $C_{28}H_{42}O_5Na$, 481.2940).

**5*β*,6*β*-epoxy-3*β*,7*α*-dihydroxy-(22*E*,24*R*)-ergosta-8(14),22-dien-15-one (3):** amorphous powder, $[\alpha]^{20.6}_{D}$ – 47.3 (*c* 0.21 MeOH); IR (KBr) $\nu_{max}$ 3430, 2958, 2932, 2871, 1627, 1437, 1382, 1224 $cm^{-1}$; UV (MeOH) $\lambda_{max}$ (logε) 253 nm (3.10), 202 nm (3.03); $^1H$ NMR data (see Table 1); $^{13}C$ NMR data (see Table 2); ESIMS (pos.) *m/z* 465 $[M + Na]^+$; HRESIMS (pos.) *m/z* 465.2980 (calcd for $C_{28}H_{42}O_4Na$, 465.2982).

**5*α*,6*α*-epoxy-3*β*,9*α*,15*α*-trihydroxy-(22*E*,24*R*)-ergosta-8(14),22-dien-7-one (4):** amorphous powder, $[\alpha]^{20.8}_{D}$ – 36.2 (*c* 0.16 MeOH); IR (KBr) $\nu_{max}$ 3440, 2958, 2931, 2871, 1732, 1661, 1627, 1583, 1461, 1384, 1266 $cm^{-1}$; UV (MeOH) $\lambda_{max}$ (logε) 267 nm (3.29), 203 nm (3.16); $^1H$ NMR data (see Table 1); $^{13}C$ NMR data (see Table 2); ESIMS (pos.) *m/z* 481 $[M + Na]^+$; HRESIMS (pos.) *m/z* 481.2929 (calcd for $C_{28}H_{42}O_5Na$,

481.2927).

**15*α*-acetoxy-(22*E*,24*R*)-ergosta-4,6,8(14),22-tetraen-3-one (5):** light yellow amorphous powder, $[\alpha]_D^{21.1}$ + 193.0 (*c* 0.09 MeOH); IR (KBr) $\nu_{max}$ 3440, 2926, 2855, 1736, 1640, 1452 cm$^{-1}$; UV (MeOH) $\lambda_{max}$ (logε) 333 nm (3.44), 203 nm (3.25); $^1$H NMR data (see Table 1); $^{13}$C NMR data (see Table 2); ESIMS (pos.) *m/z* 473 [M + Na]$^+$; HRESIMS (pos.) *m/z* 451.3212 (calcd for $C_{30}H_{42}O_3H$, 451.3221).

**15*β*-methoxy-(22*E*,24*R*)-ergosta-4,6,8(14),22-tetraen-3-one (6):** light yellow amorphous powder, $[\alpha]_D^{20.4}$ + 231.8 (*c* 0.09 MeOH); IR (KBr) $\nu_{max}$ 3440, 2926, 2855, 1736, 1640, 1452 cm$^{-1}$; UV (MeOH) $\lambda_{max}$ (logε) 336 nm (3.21), 221 nm (3.45), 201 nm (3.45),; $^1$H NMR data (see Table 1); $^{13}$C NMR data (see Table 2); ESIMS (pos.) *m/z* 445 [M + Na]$^+$; HRESIMS (pos.) *m/z* 423.3263 (calcd for $C_{29}H_{42}O_2H$, 423.3262).

## Electronic Supplementary Material

Supplementary material is available in the online version of this article at http://dx.doi.org/ 10.1007/s13659-012-0058-4 and is accessible for authorized users.

## Acknowledgments

This work was financially supported by National Basic Research Program of China (973 Program, 2009CB522300), the National Natural Science Foundation of China (30830113, U1132607).

**Open Access** This article is distributed under the terms of the Creative Commons Attribution License which permits any use, distribution, and reproduction in any medium, provided the original author(s) and source are credited.

## Electronic supplementary material

Supplementary material, approximately 6.54 MB.

## References

[1] Gao J. M., Zhang A. L., Yao H. Y. (2003). Zhongguo Zhongyao Zazhi.

[2] Gao J. M., Hu L., Dong Z. J., Liu J. K. (2001). Lipids.

[3] Liu J. K. (2002). Heterocycles.

[4] Wang, F. Z.; Fang, Y. C.; Zhang, M.; Gu, Q. Q.; Zhu, W. M. *Steroids* **2008**, 73, 19–26. 10.1016/j.steroids.2007.08.008 17900642

[5] Taro A., Makoto T., Atsushi N. (2007). J. Nat. Prod..

[6] Ponce M. A., Ramirez J. A., Erra-Balsells R. (2002). Photochem. Photobiol. Sci..

[7] Adler J. H., Young M., Nes W. R. (1977). Lipids.

[8] Wright J. L. C., McInnes A. G., Shimizu S. (1978). Can. J. Chem..

[9] John Wiley & Sons: Chichester, West Sussex, England, **2002**, p 232.

[10] Tayyab A. M., Lee Y. M., Jung J. H. (2006). J. Nat. Prod..

[11] Bridgeman, J. E.; Cherry, P. C.; Woodgate, P. D. *J. Chem. Soc.* **1970**, 250–257. 10.1039/j39700000250 5460858

[12] Schulte K. E., Buecker G., Fachmann H. (1968). Tetrahedron Lett..

