# Supplementary material for: Six novel steroids from culture of basidiomycete Polyporus ellisii
Source: Nat Prod Bioprospect. 2012 Dec 12;2(6):240–4. doi: 10.1007/s13659-012-0058-4 (PMC4131608; doi:10.1007/s13659-012-0058-4)

Electronic Supplementary Material

Six Novel Steroids from Culture of Basidiomycete *Polyporus ellisii*

Shuang WANG,[a,b] Ling ZHANG,[a] Liang-Yan LIU,[a,b] Ze-Jun DONG,[a] Zheng-Hui LI,[a] and Ji-Kai LIU[a,]*

[a]State Key Laboratory of Phytochemistry and Plant Resources in West China, Kunming Institute of Botany, Chinese Academy of Sciences, Kunming 650201, China

[b]University of Chinese Academy of Sciences, Beijing 100049, China

Received 17 July 2012; Accepted 8 October 2012

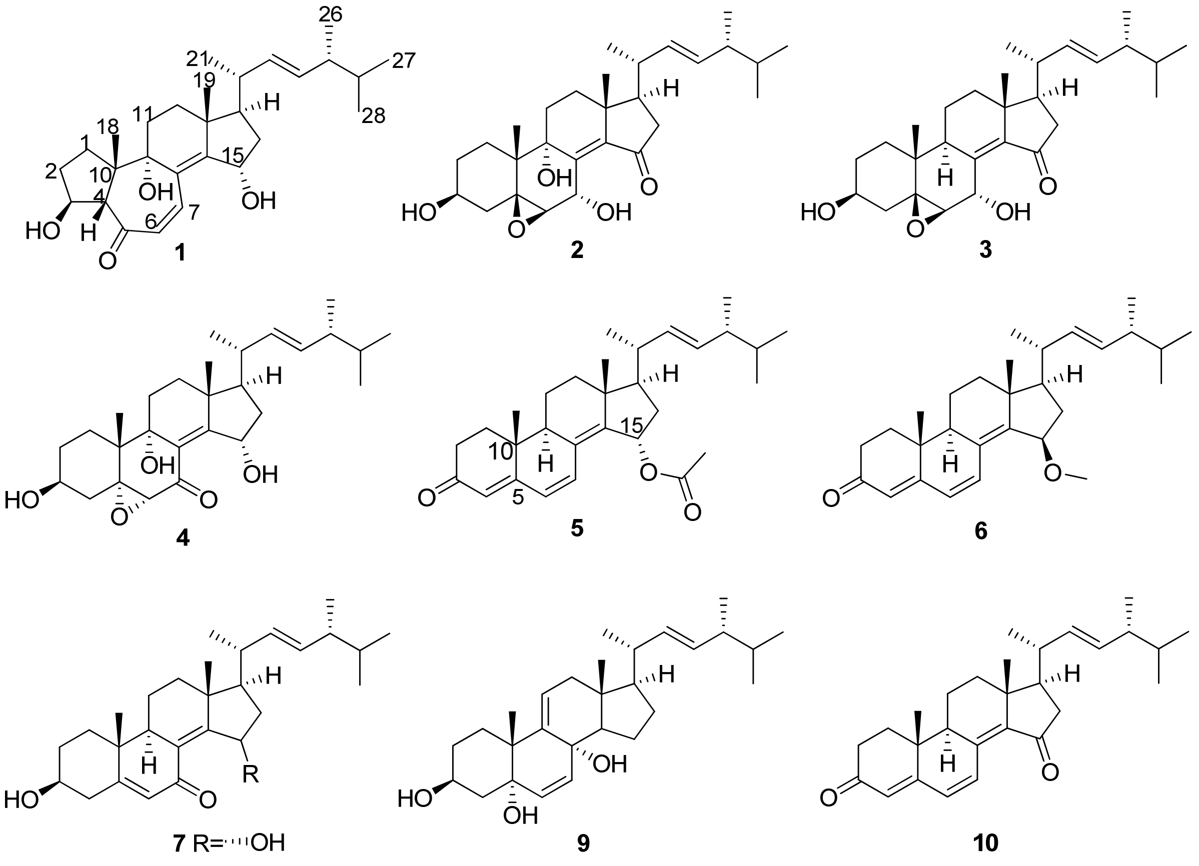

Structures of compounds **1**–**10**

*To whom correspondence should be addressed. E-mail: jkliu@mail.kib.ac.cn

^{1}H NMR spectrum of **1** in $CDCl_3$.

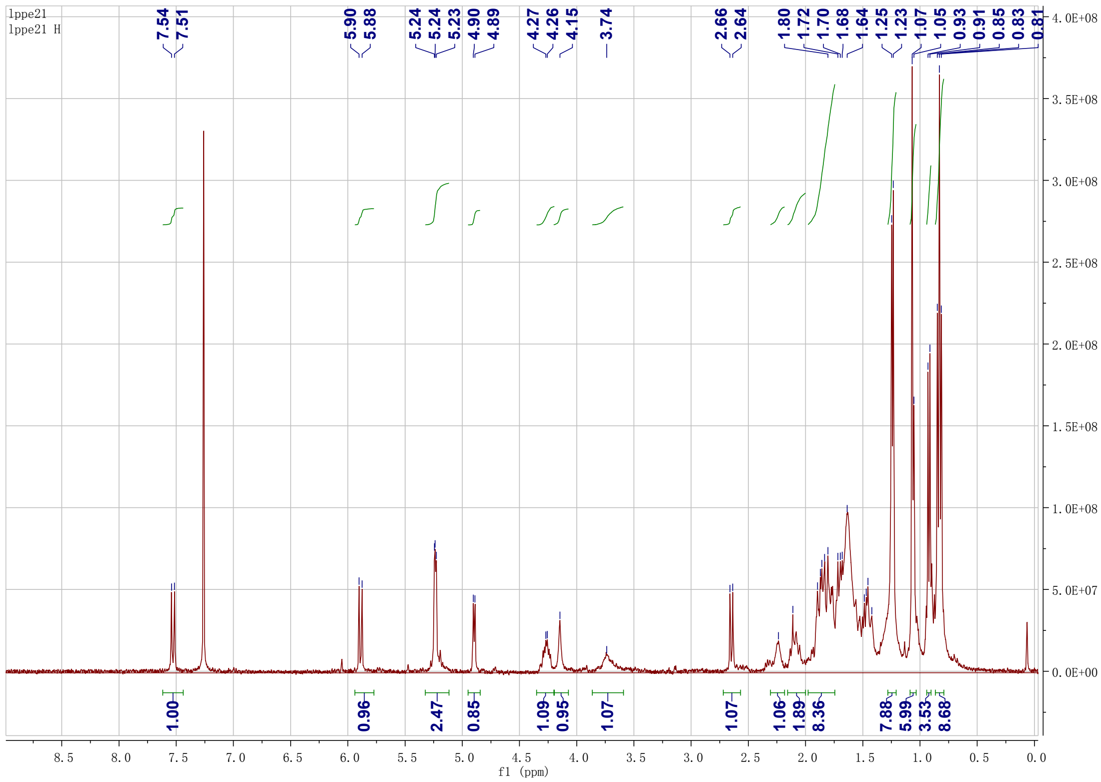

^{13}C NMR and DEPT spectra of **1** in $CDCl_3$.

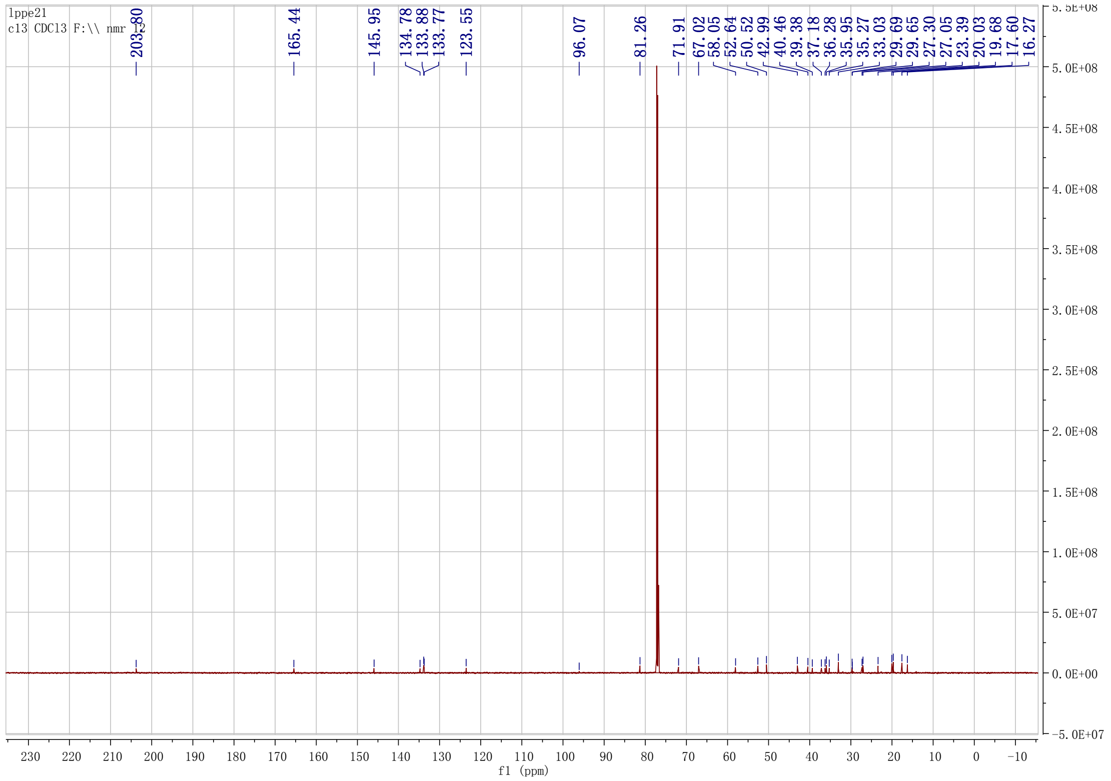

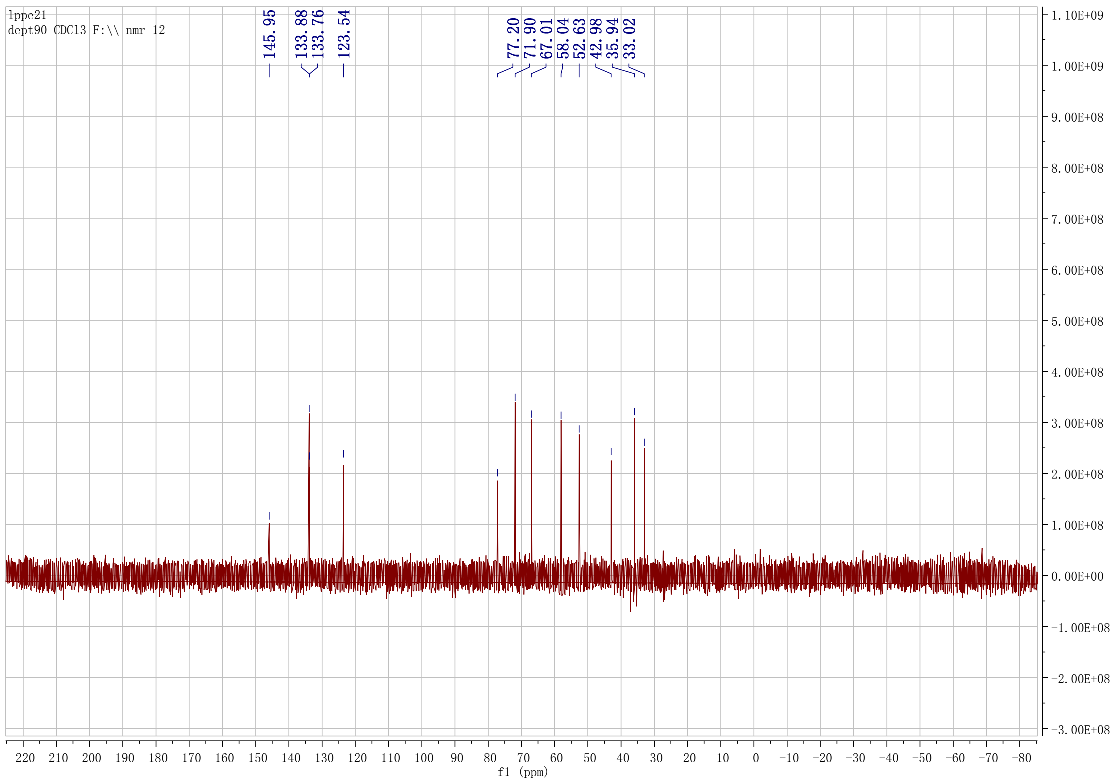
lppe21
dept90 CDCl3 F:\\ nmr 12
145.95
133.88
133.76
123.54
77.20
71.90
67.01
58.04
52.63
42.98
35.94
33.02
f1 (ppm)

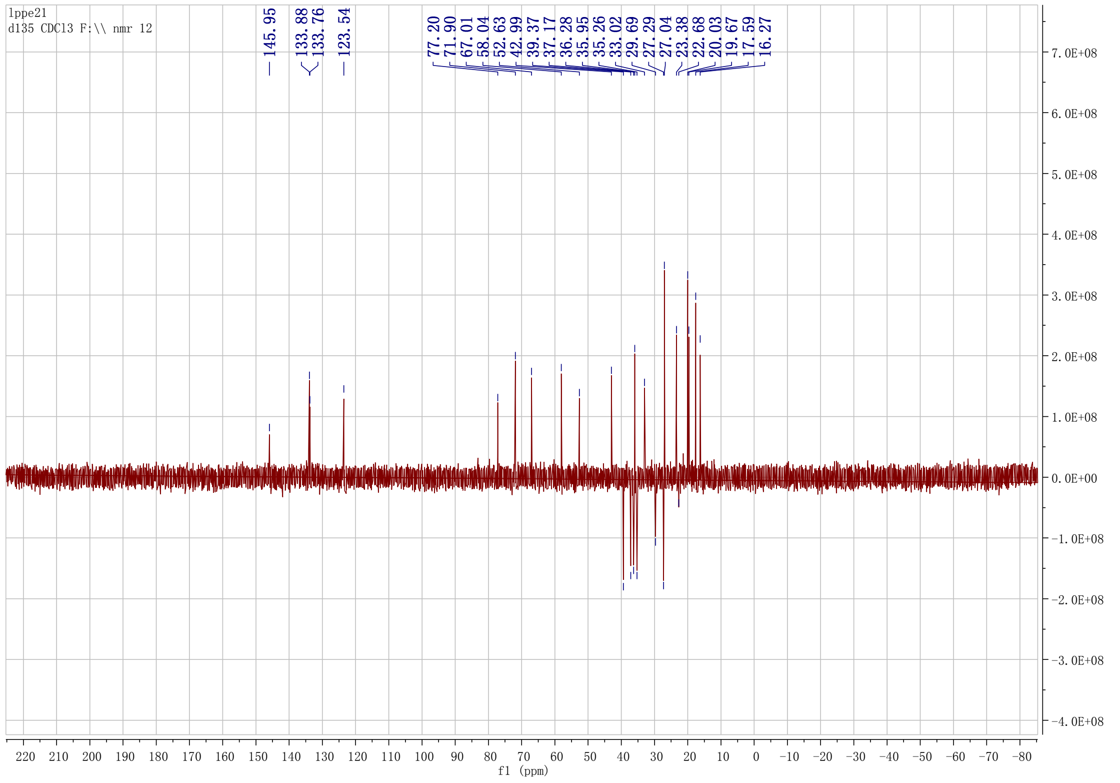
lppe21
d135 CDCl3 F:\\ nmr 12
145.95
133.88
133.76
123.54
77.20
71.90
67.01
58.04
52.63
42.99
39.37
37.17
36.28
35.95
35.26
33.02
29.69
27.29
27.04
23.38
22.68
20.03
19.67
17.59
16.27
f1 (ppm)

HSQC of **1**.

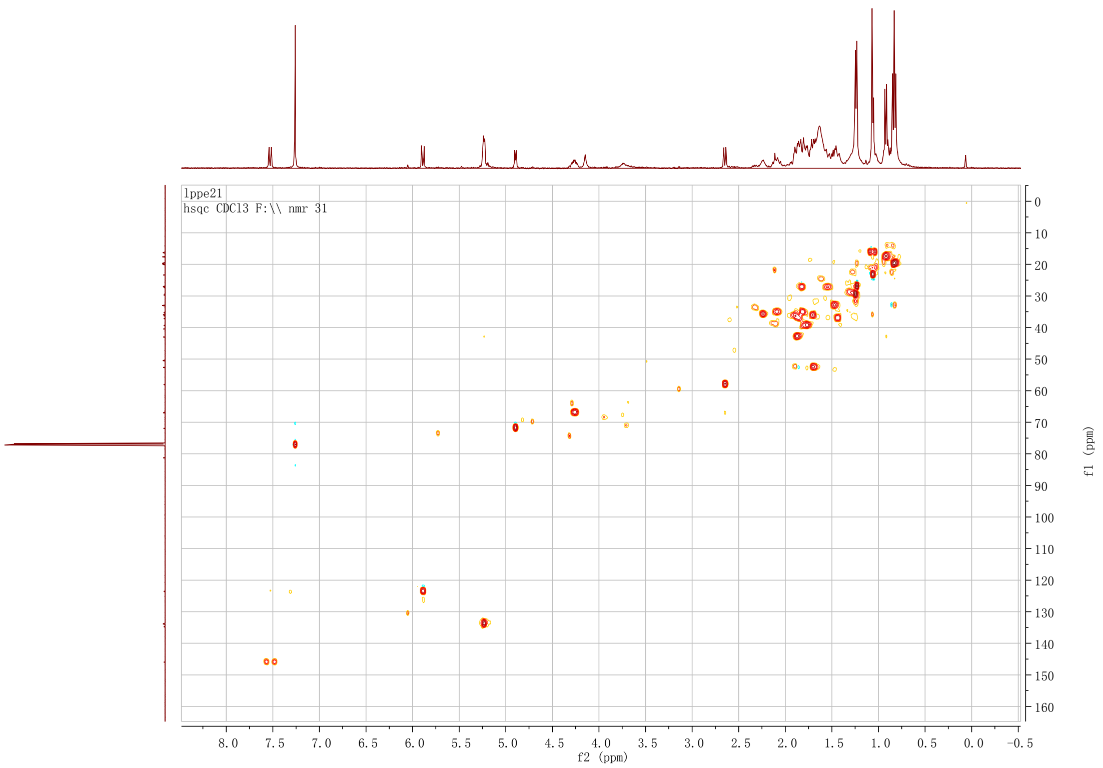

HMBC of **1**.

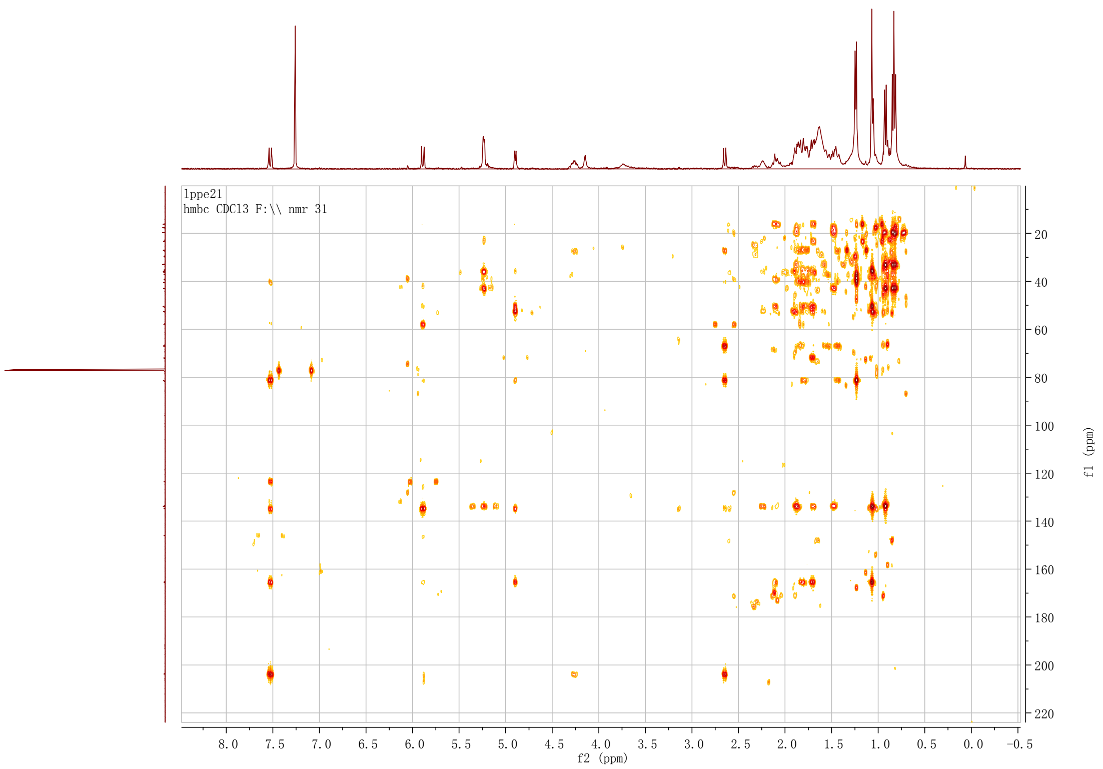

ROESY of **1**.

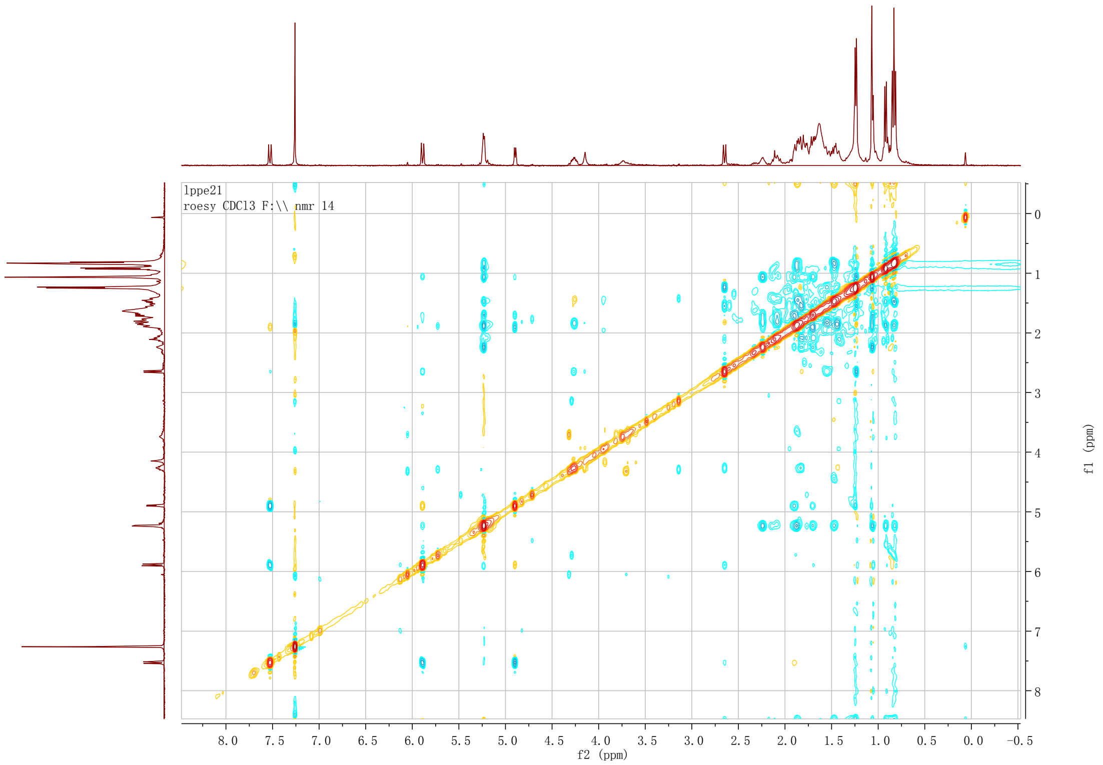

COSY of **1**.

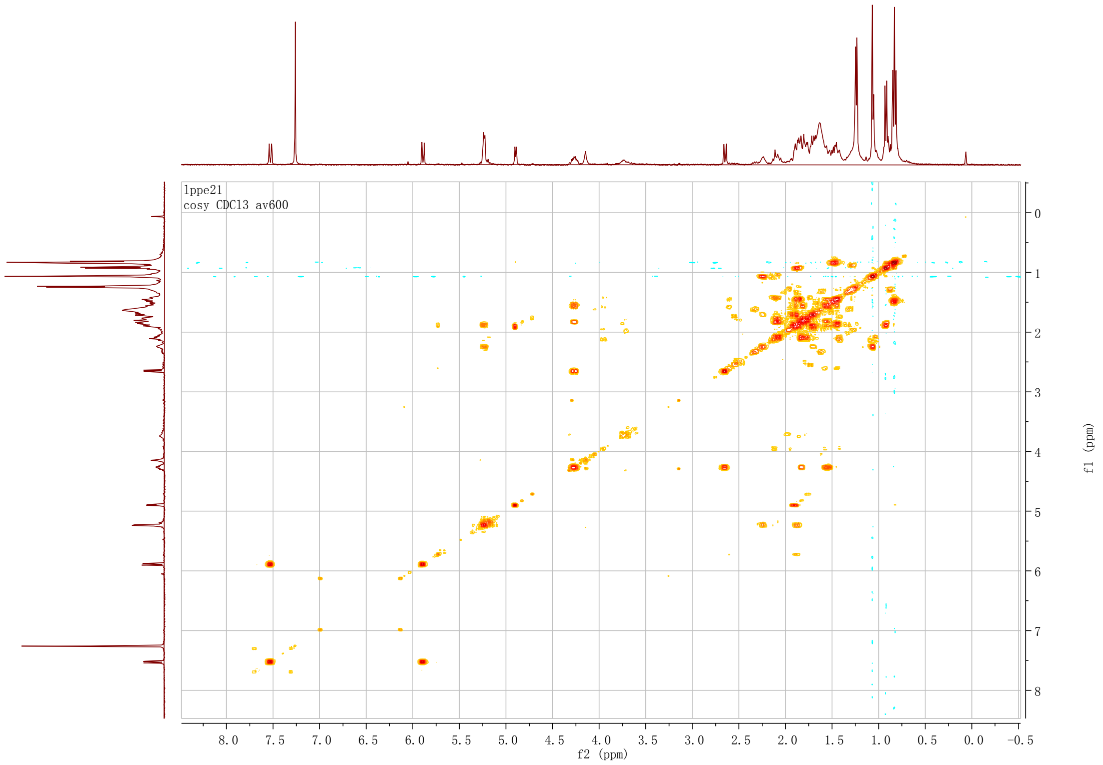

HRESIMS spectrum of **1**.

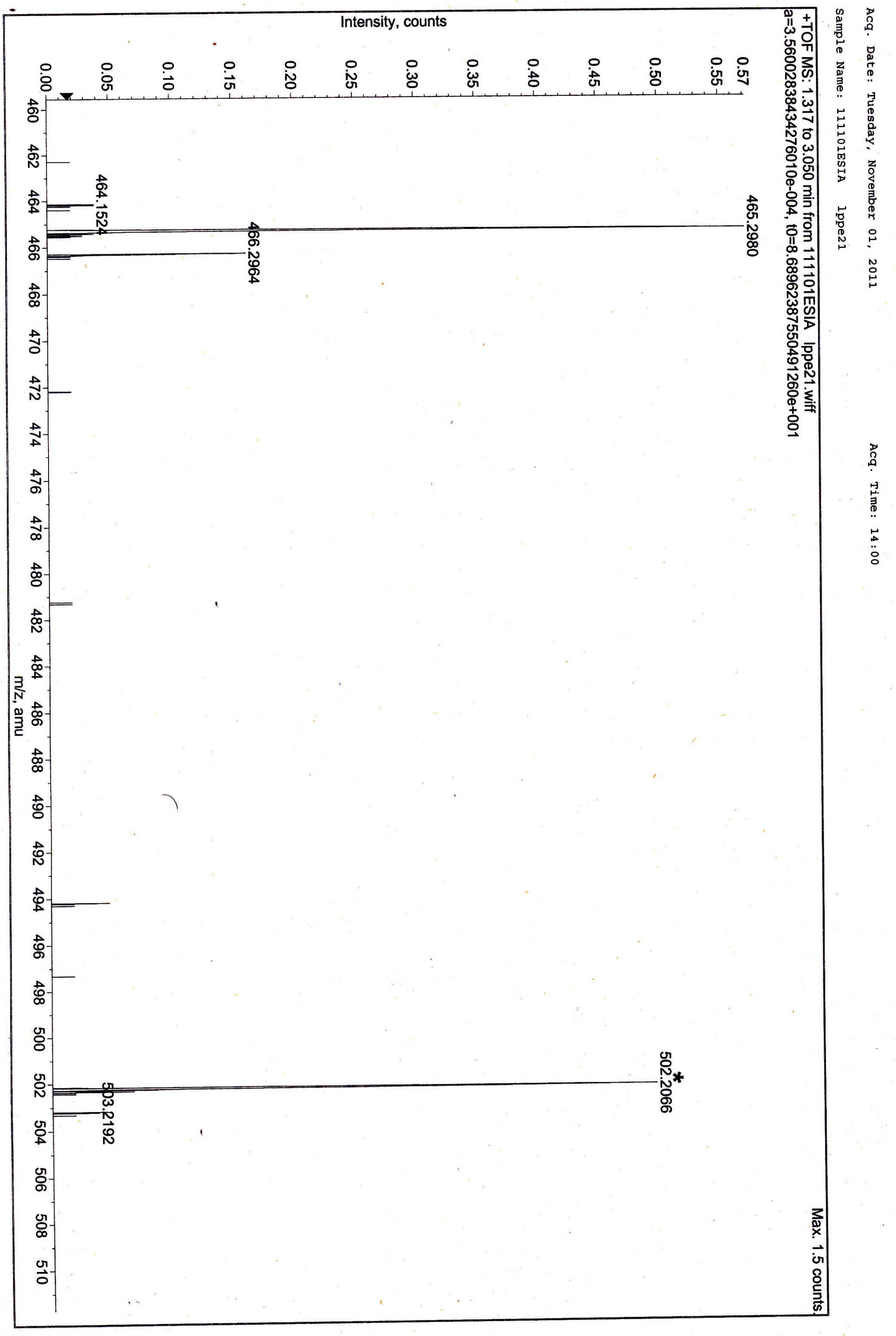

^{1}H NMR spectrum of **1** in DMSO.

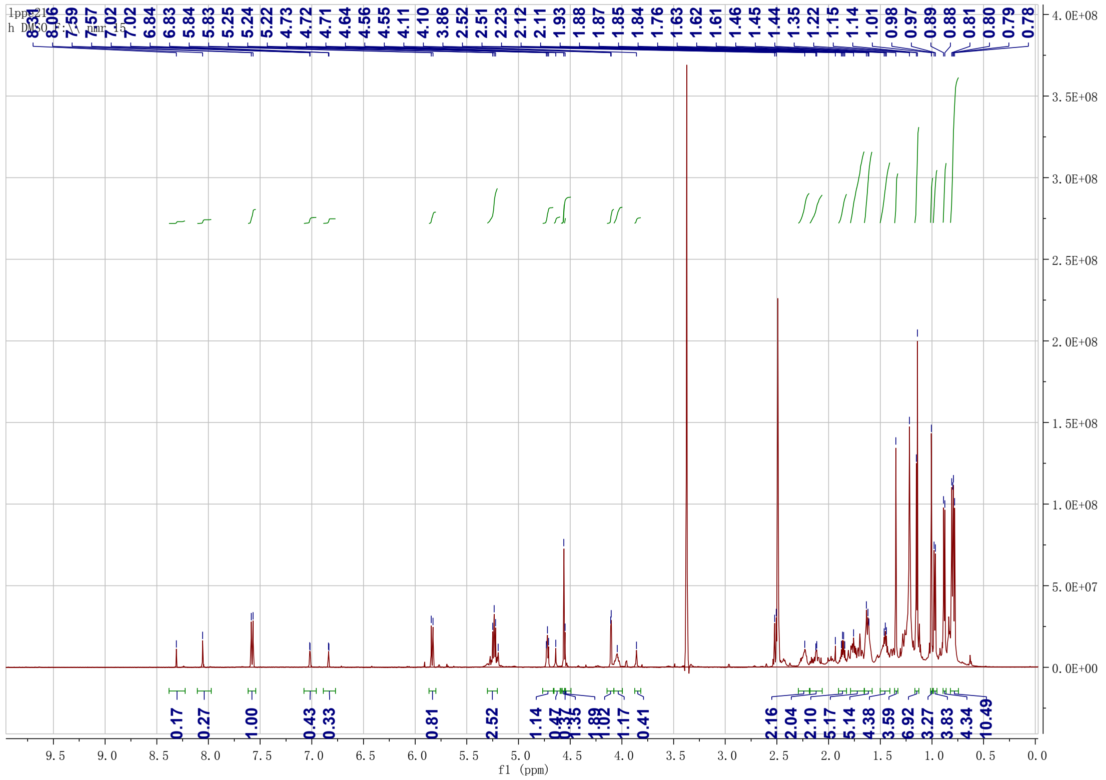

ROESY of **1** in DMSO.

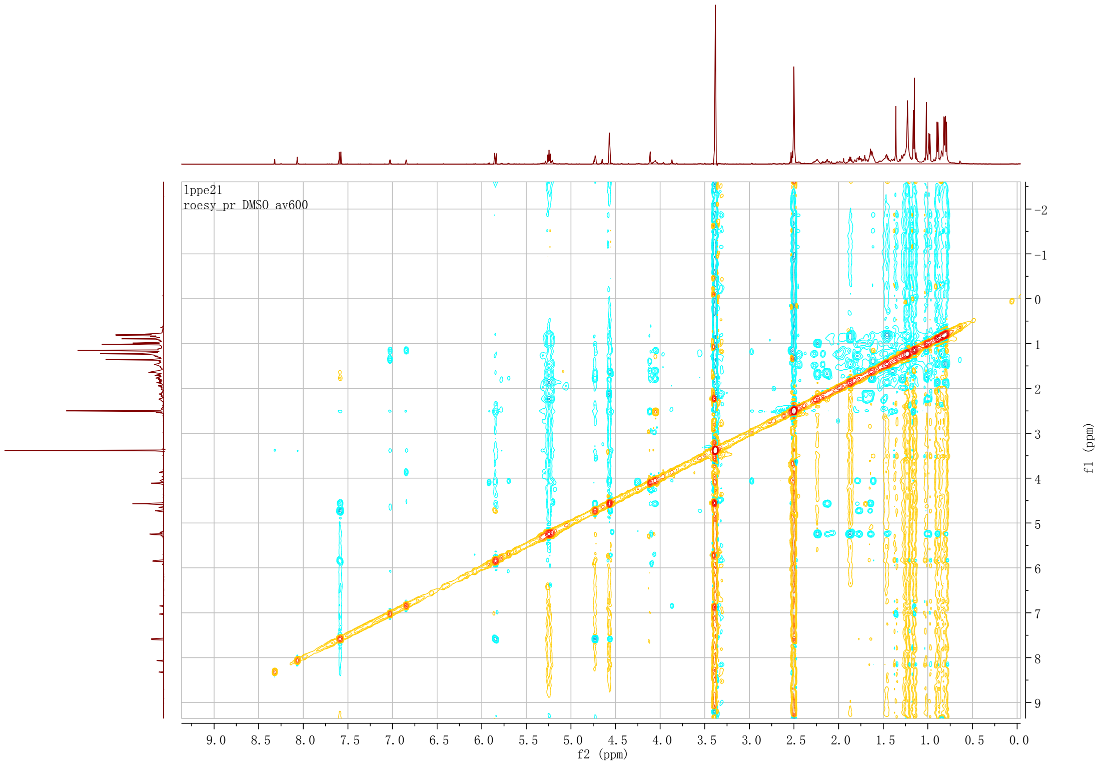

^{1}H NMR spectrum of **2** in $CDCl_3$.

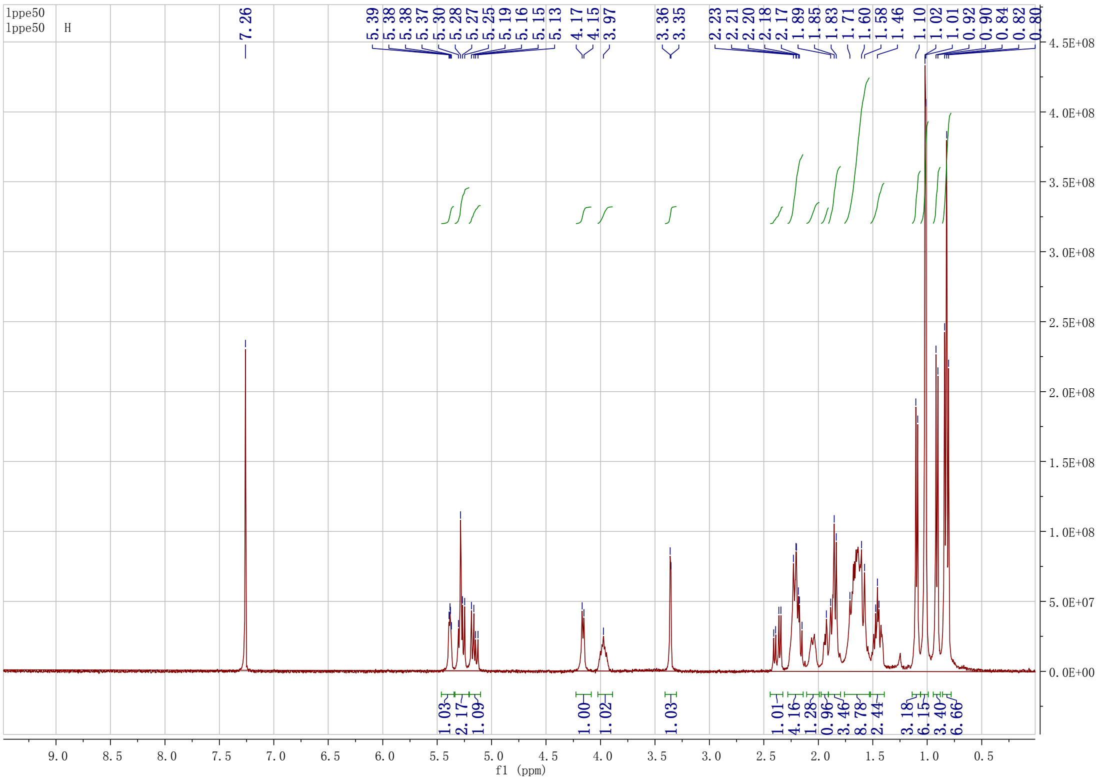

^{13}C NMR and DEPT spectra of **2** in $CDCl_3$.

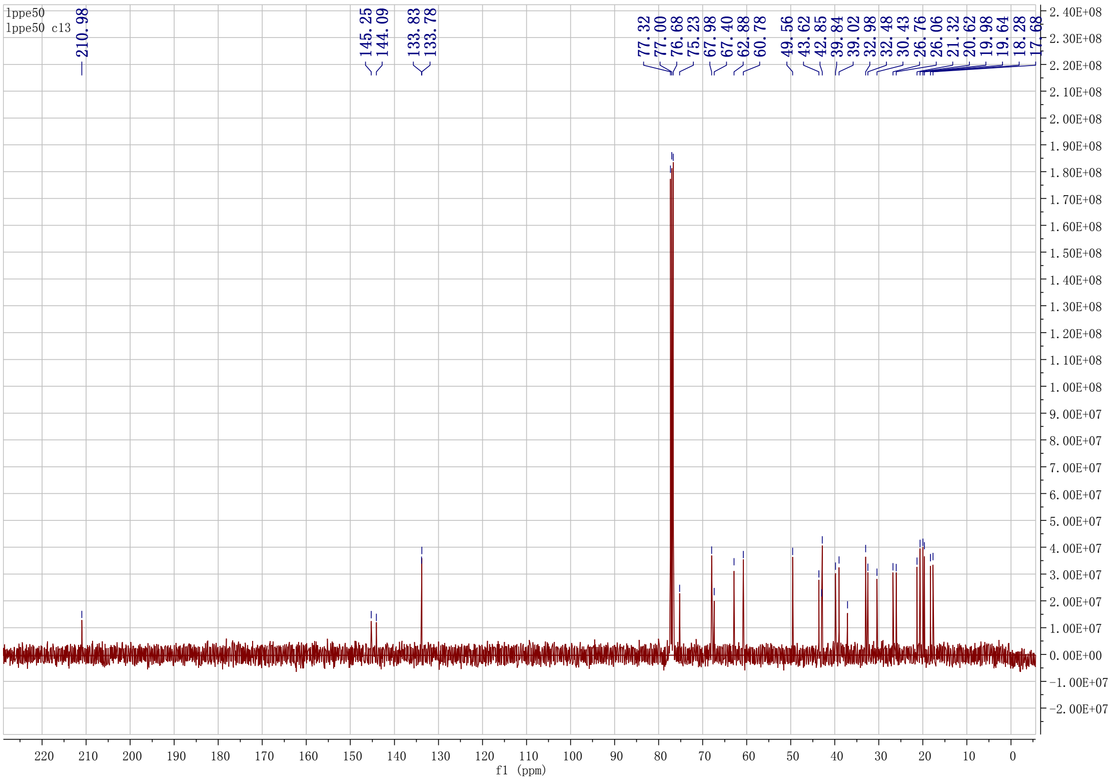

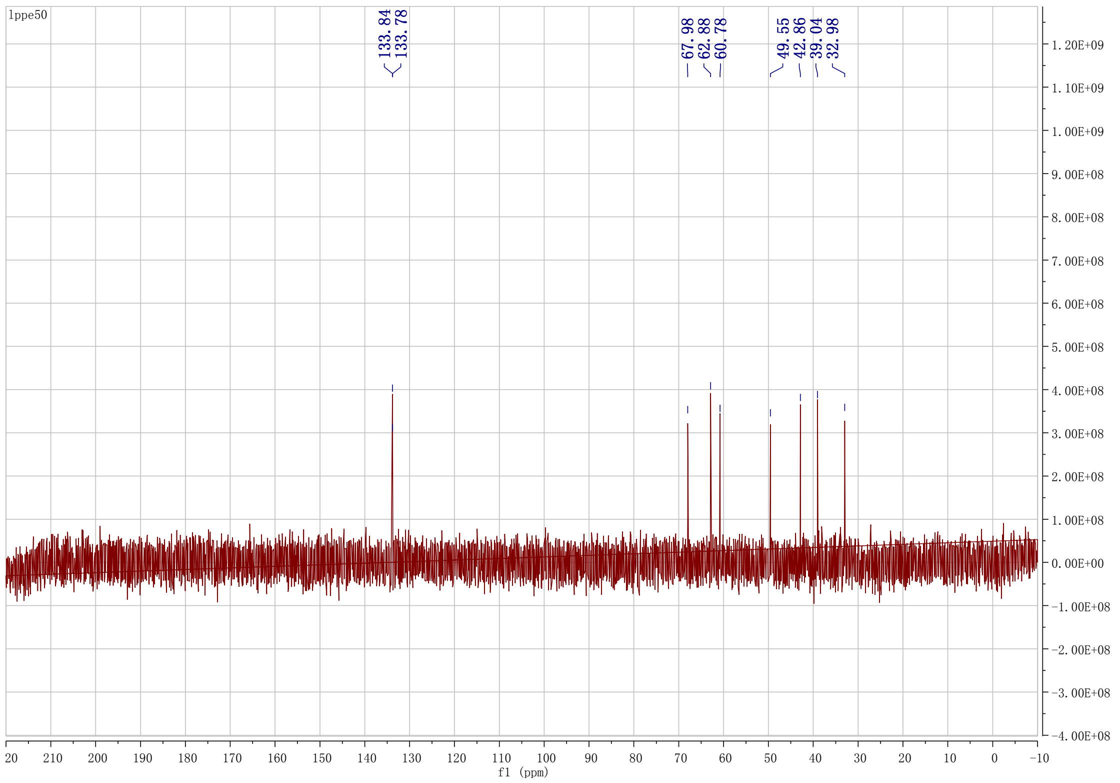
lppe50
133.84
133.78
67.98
62.88
60.78
49.55
42.86
39.04
32.98
f1 (ppm)

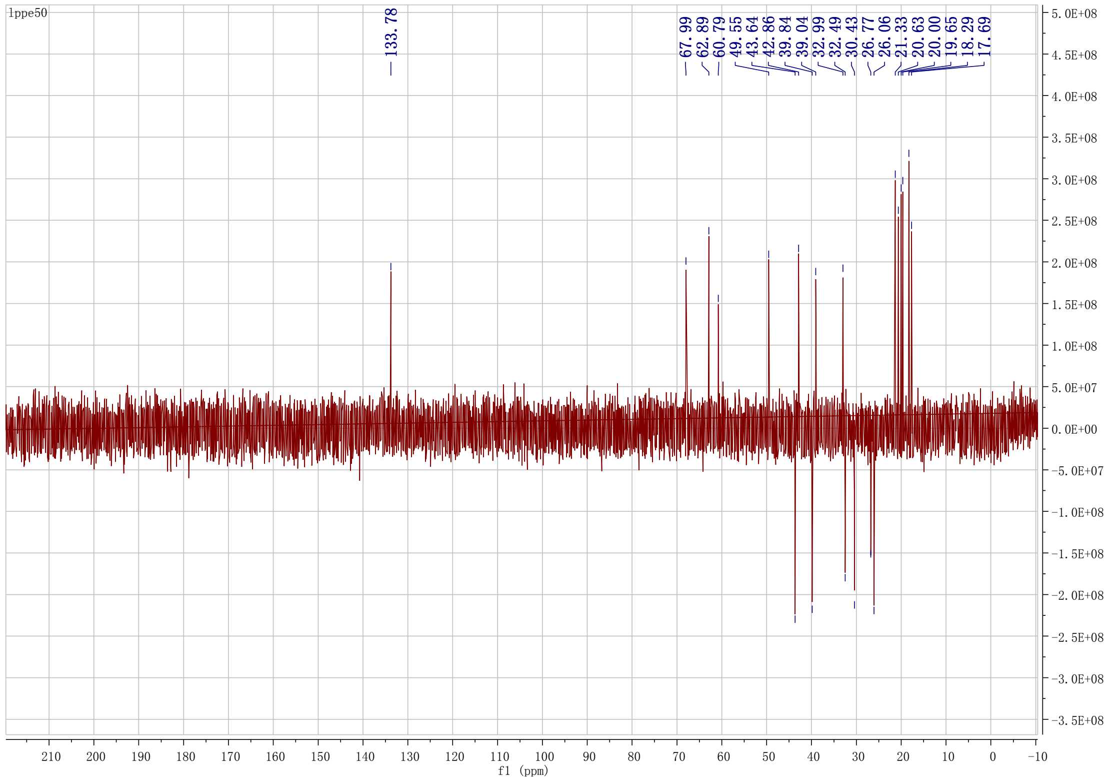
lppe50
133.78
67.99
62.89
60.79
49.55
43.64
42.86
39.84
39.04
32.99
32.49
30.43
26.77
26.06
21.33
20.63
20.00
19.65
18.29
17.69
f1 (ppm)

HSQC of **2**.

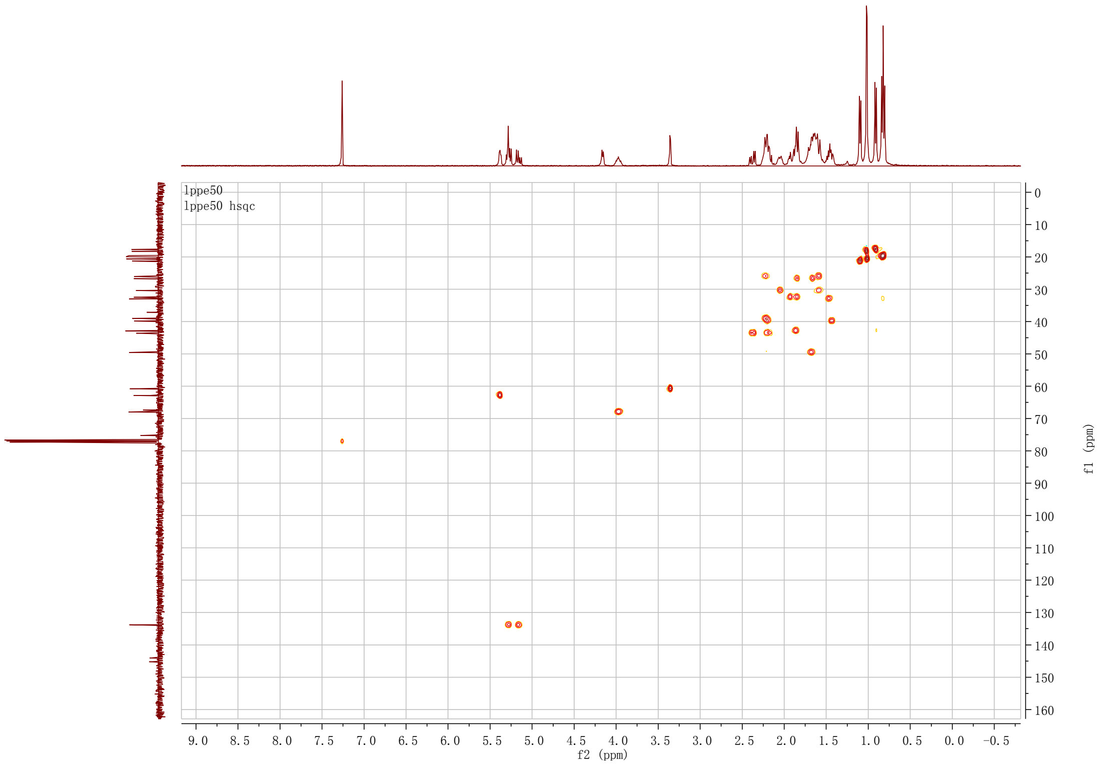

HMBC of **2**.

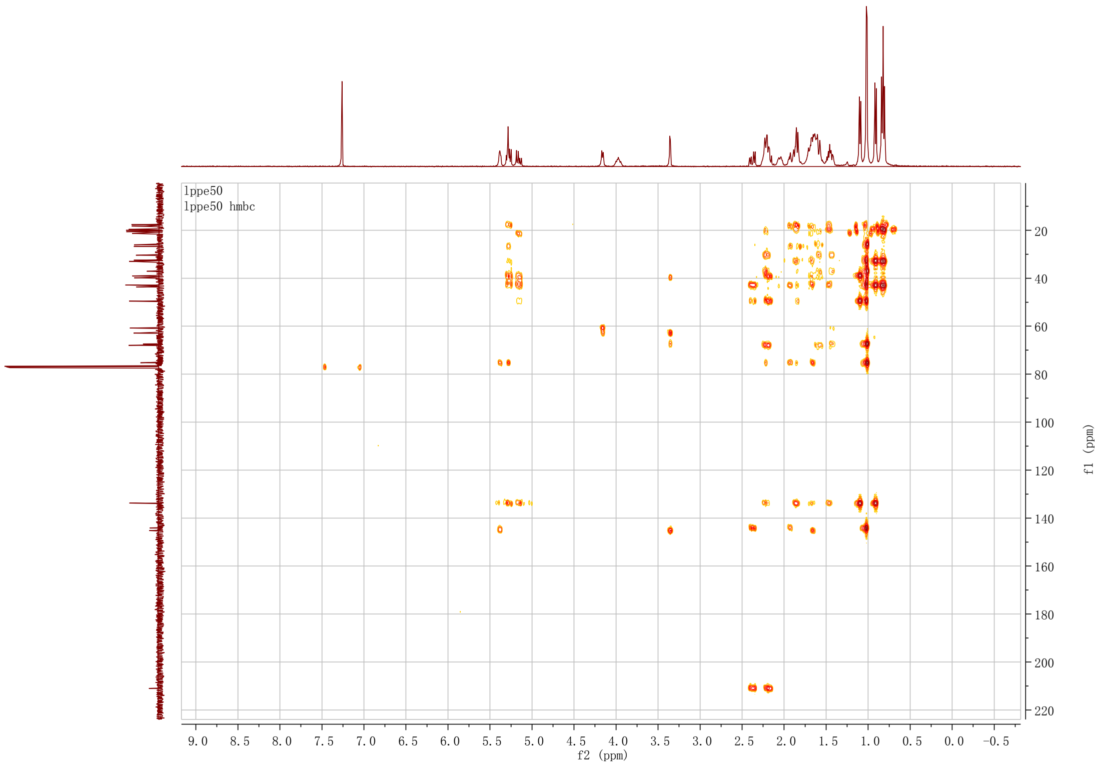

ROESY of **2**.

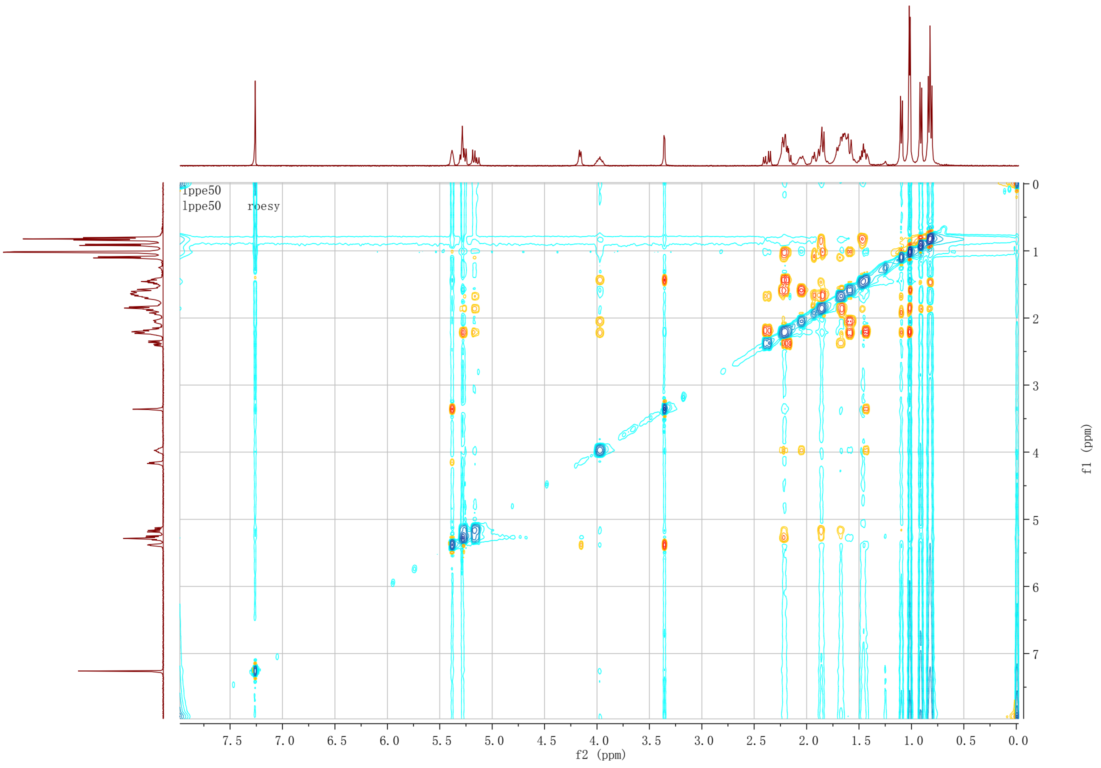

HRESIMS spectrum of **2**.

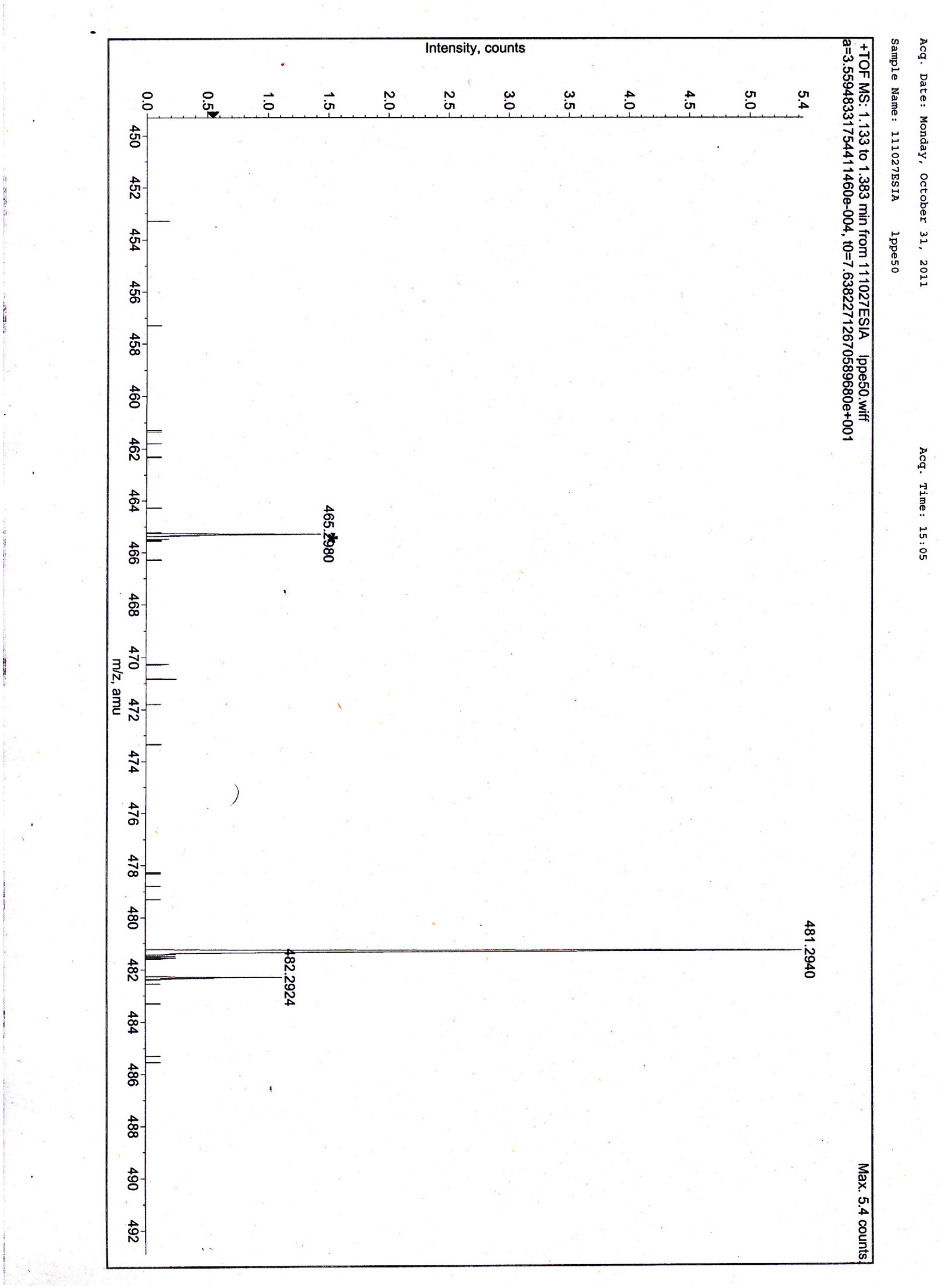

^{1}H NMR spectrum of **2** in DMSO.

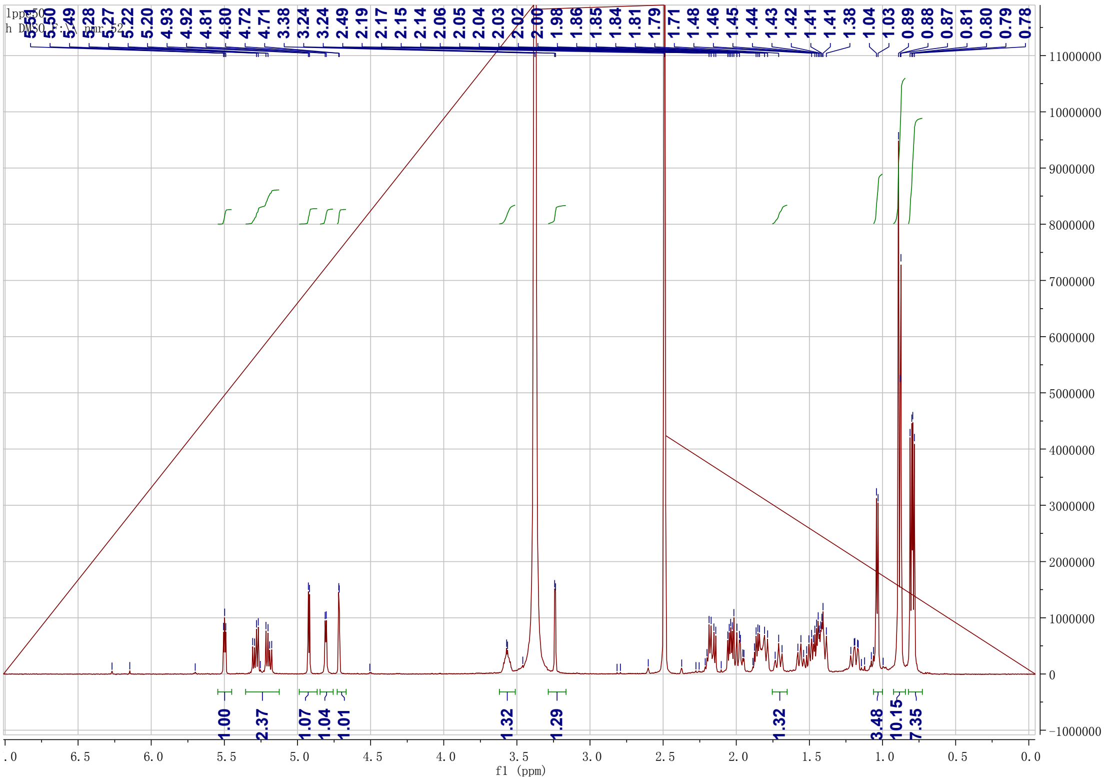

ROESY of **2** in DMSO.

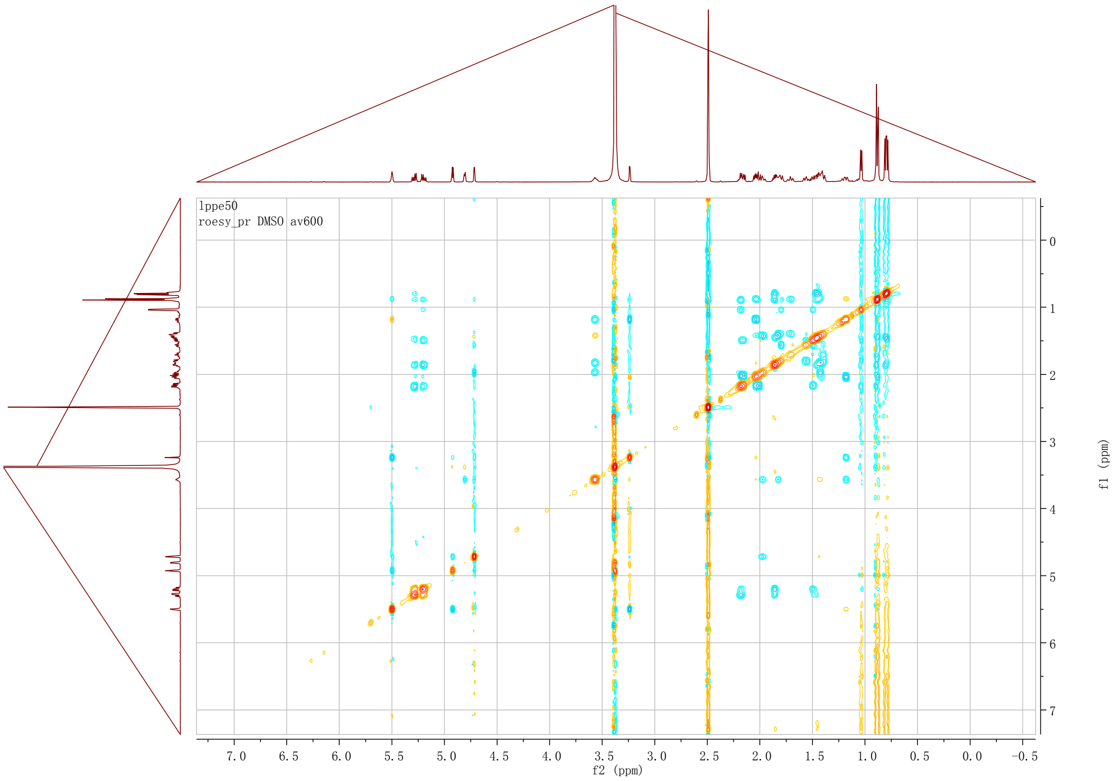

^{1}H NMR spectrum of **3** in $CDCl_3$.

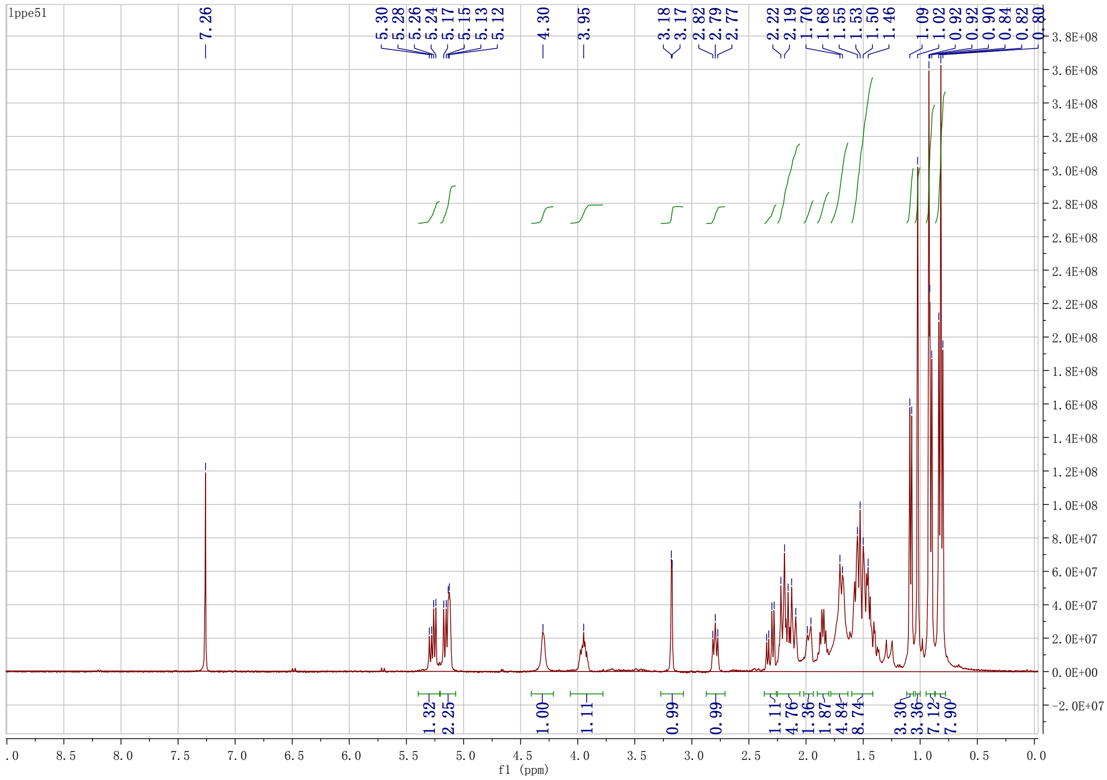

^{13}C NMR and DEPT spectra of **3** in $CDCl_3$.

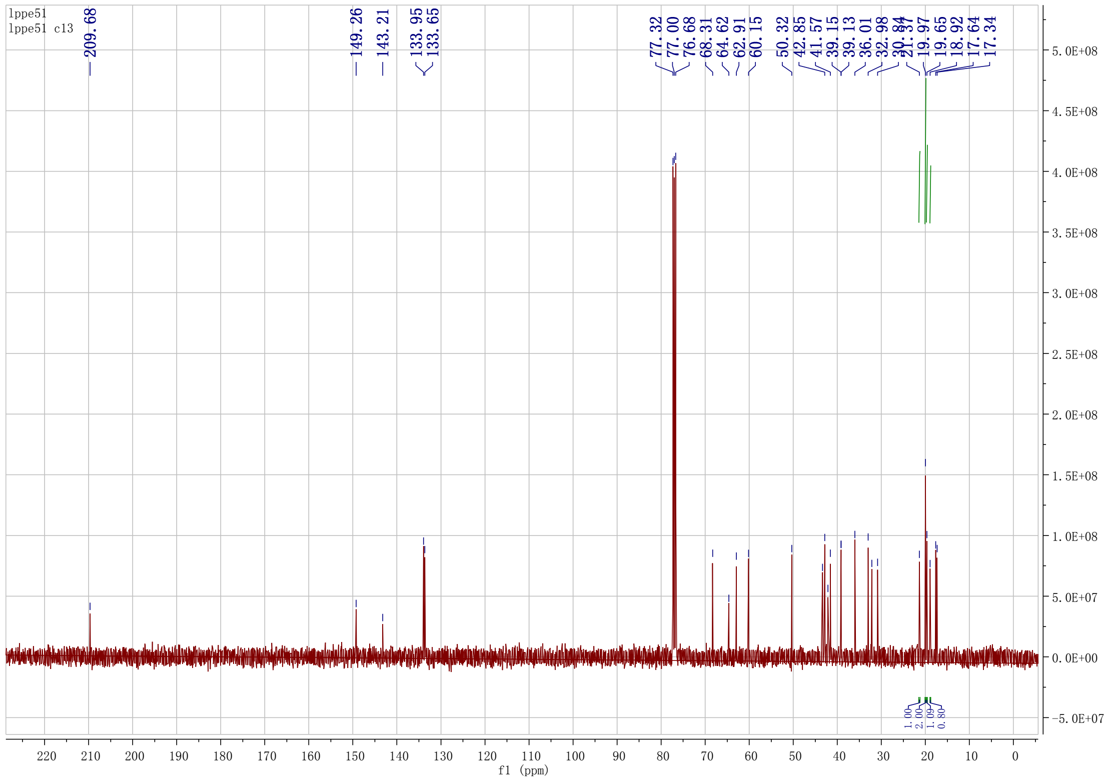

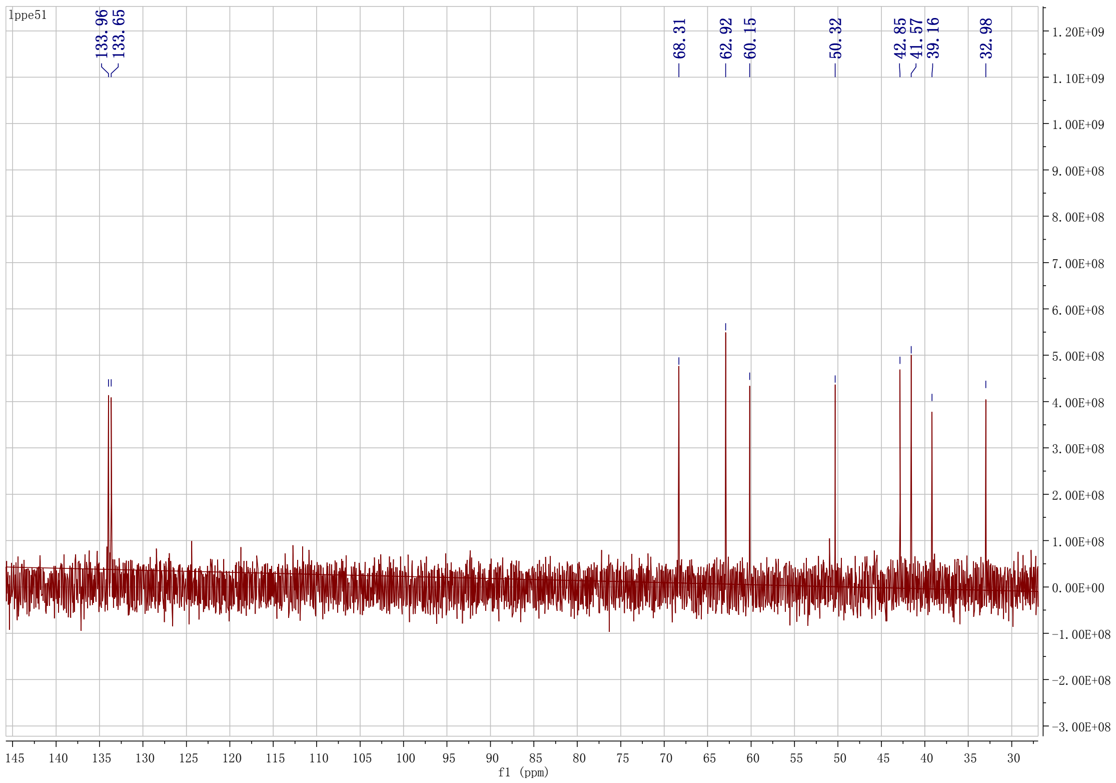
lppe51
133.96
133.65
68.31
62.92
60.15
50.32
42.85
41.57
39.16
32.98
f1 (ppm)

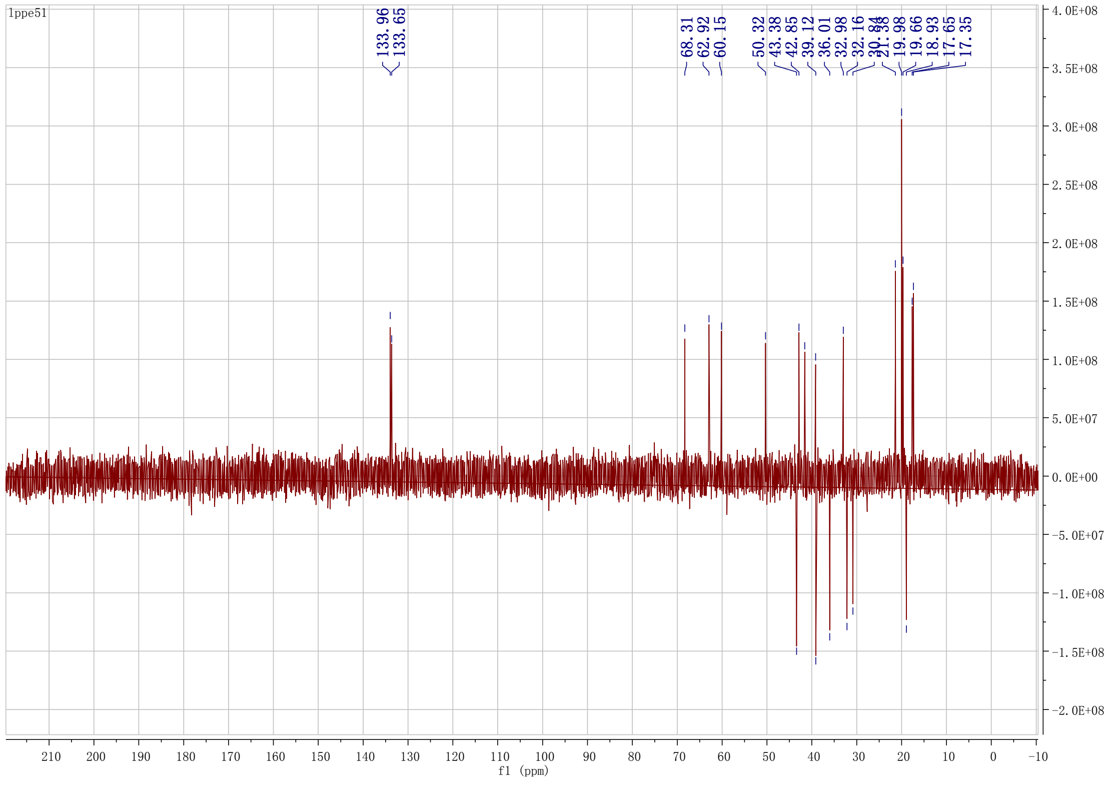
lppe51
133.96
133.65
68.31
62.92
60.15
50.32
43.38
42.85
39.12
36.01
32.98
32.16
19.98
19.66
18.93
17.65
17.35
f1 (ppm)

HSQC of **3**.

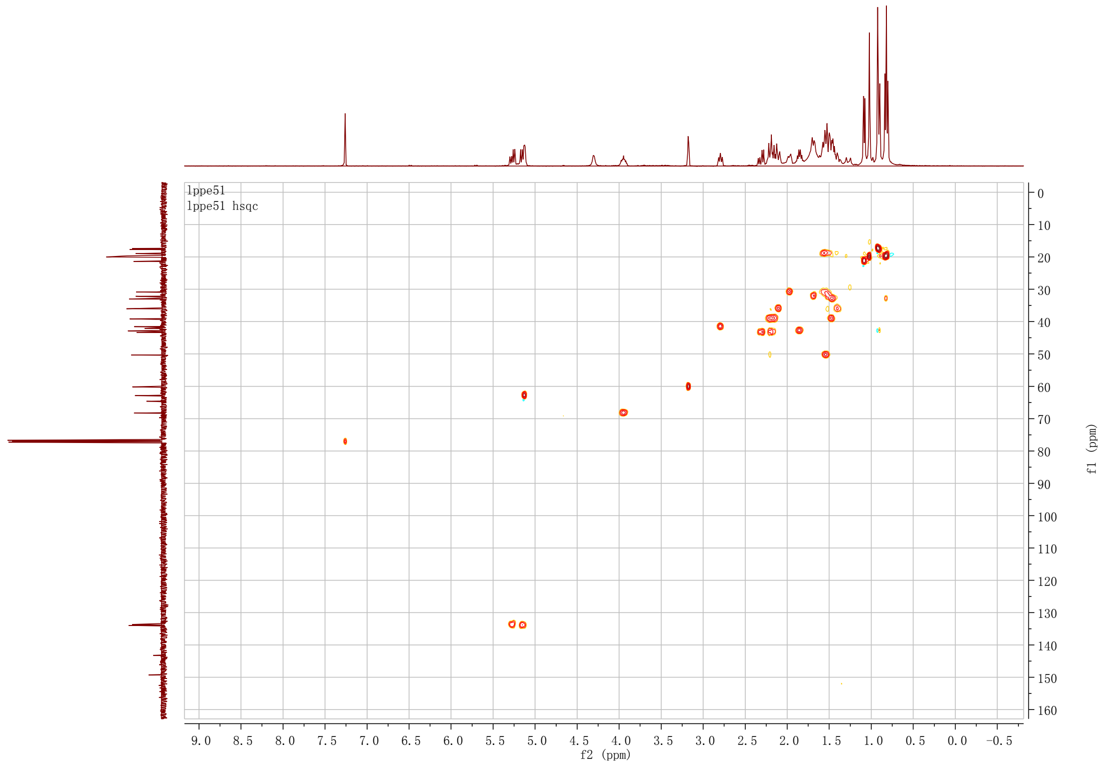

HMBC of **3**.

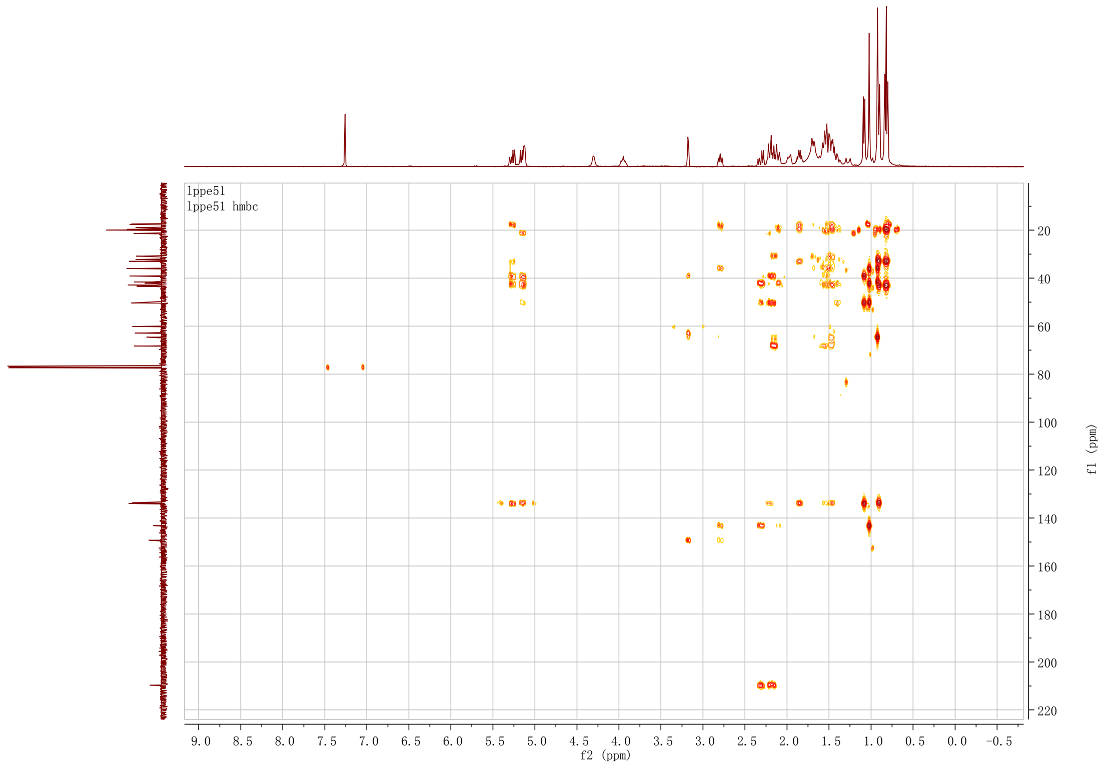

ROESY of **3**.

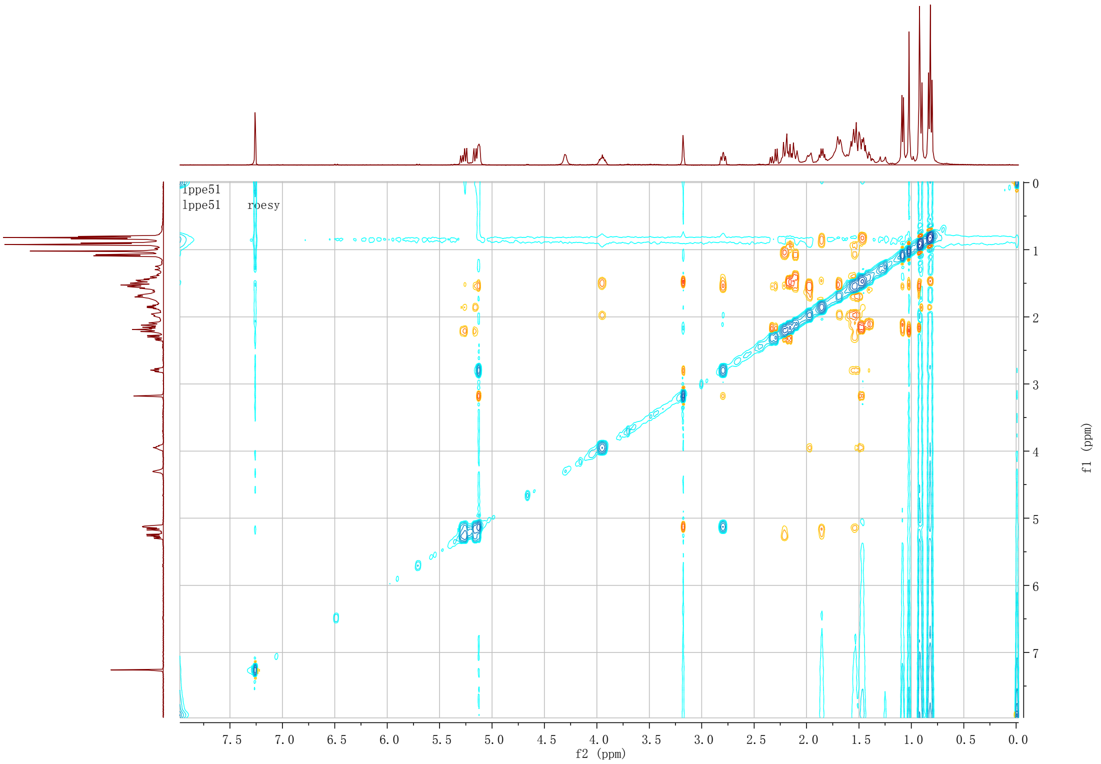
lppe51
lppe51 roesy
f2 (ppm)
f1 (ppm)
7.5
7.0
6.5
6.0
5.5
5.0
4.5
4.0
3.5
3.0
2.5
2.0
1.5
1.0
0.5
0.0
0
1
2
3
4
5
6
7

HRESIMS spectrum of **3**.

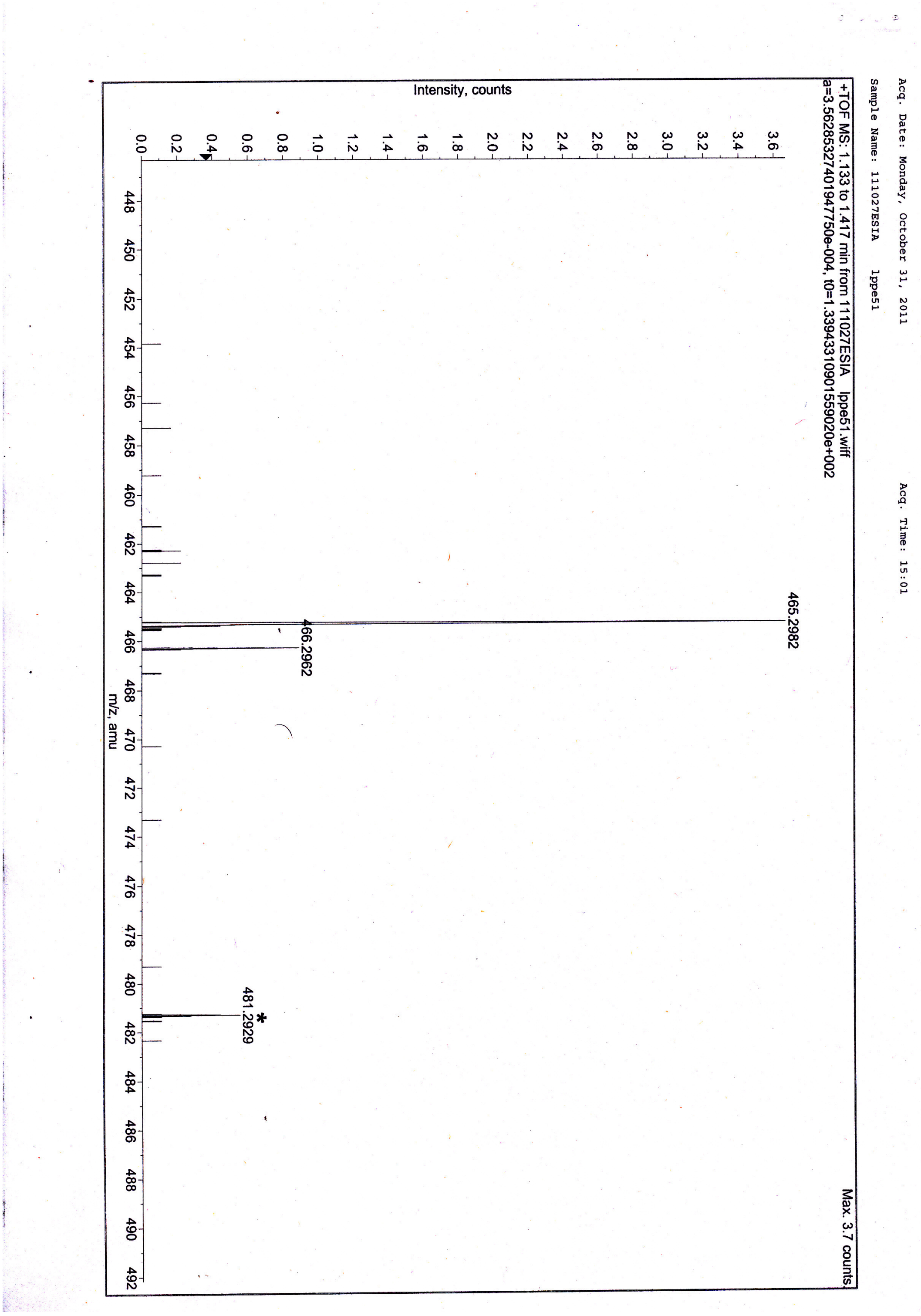

^{1}H NMR spectrum of **4** in $CDCl_3$.

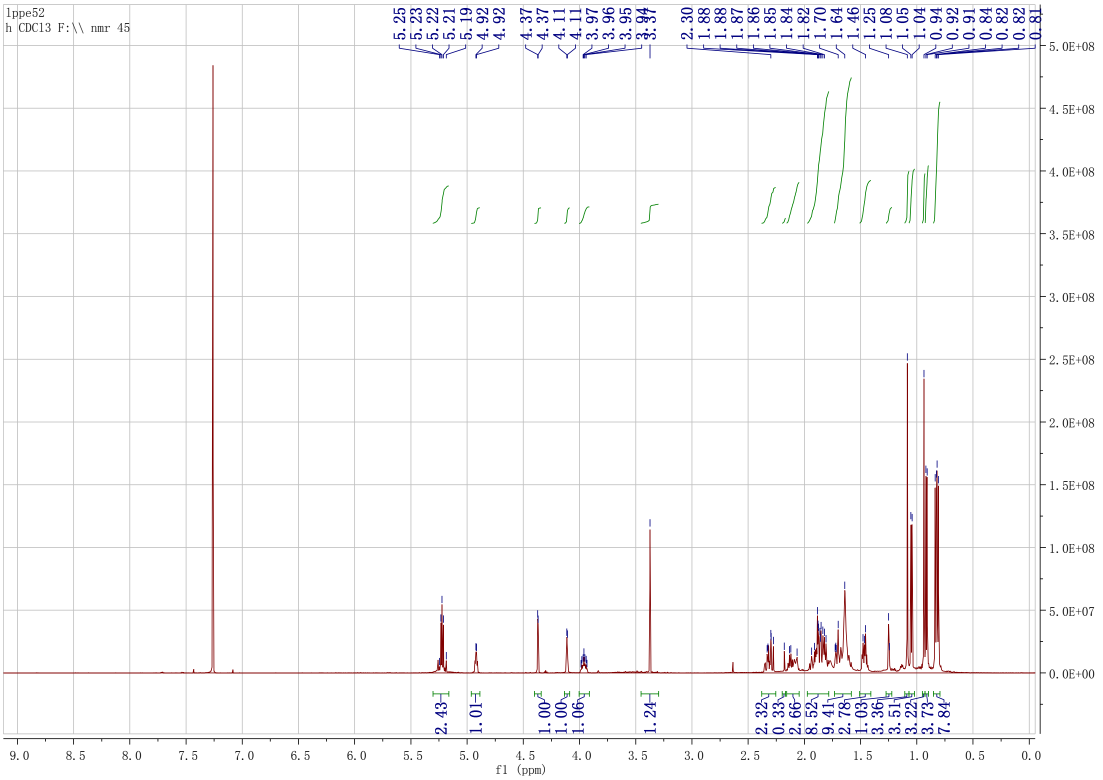

^{13}C NMR and DEPT spectra of **4** in $CDCl_3$.

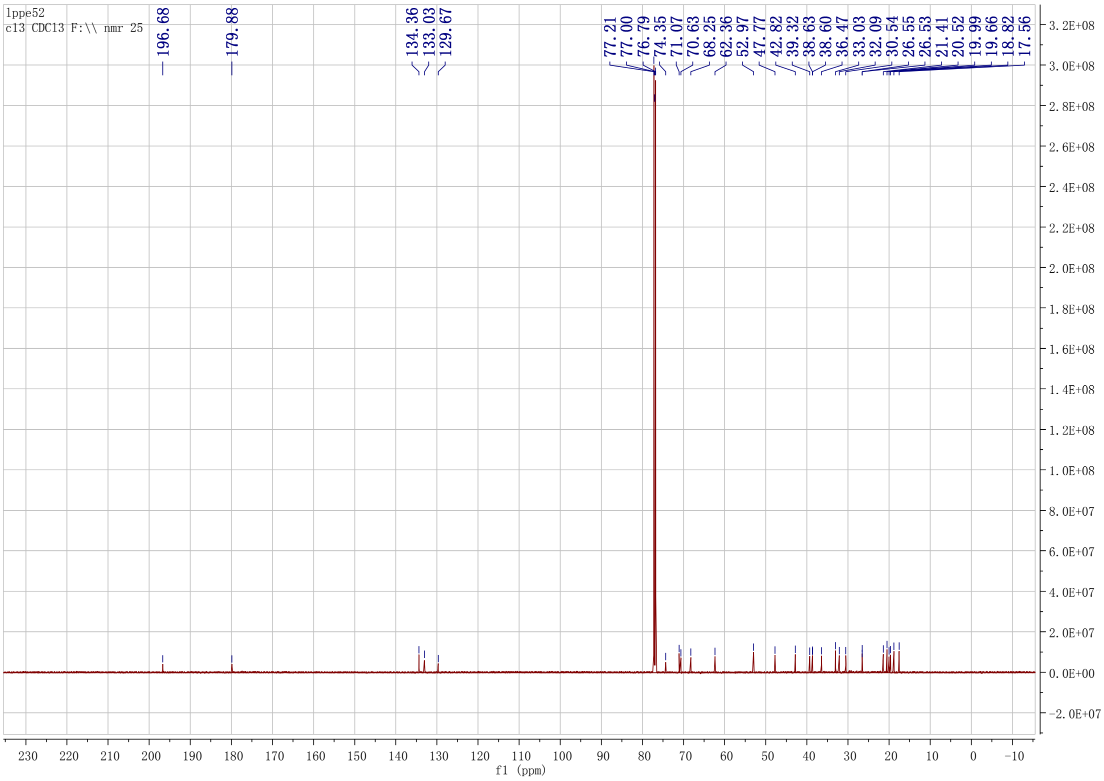

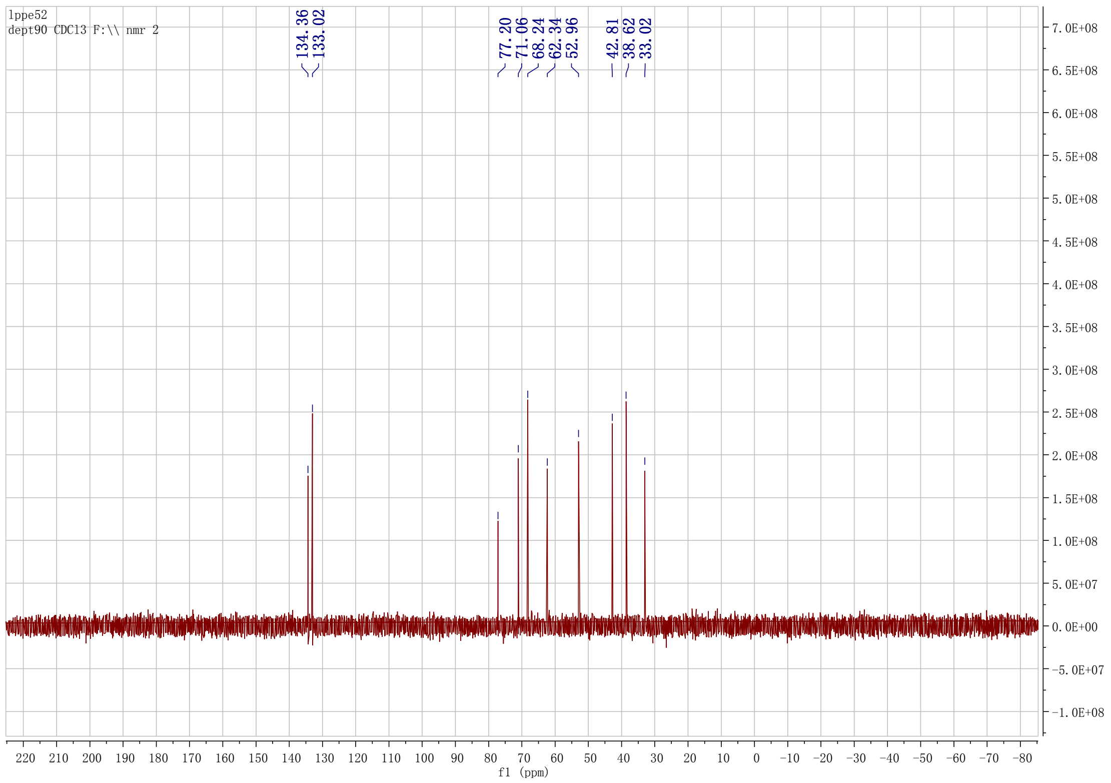
lppe52
dept90 CDCl3 F:\\ nmr 2
134.36
133.02
77.20
71.06
68.24
62.34
52.96
42.81
38.62
33.02
f1 (ppm)

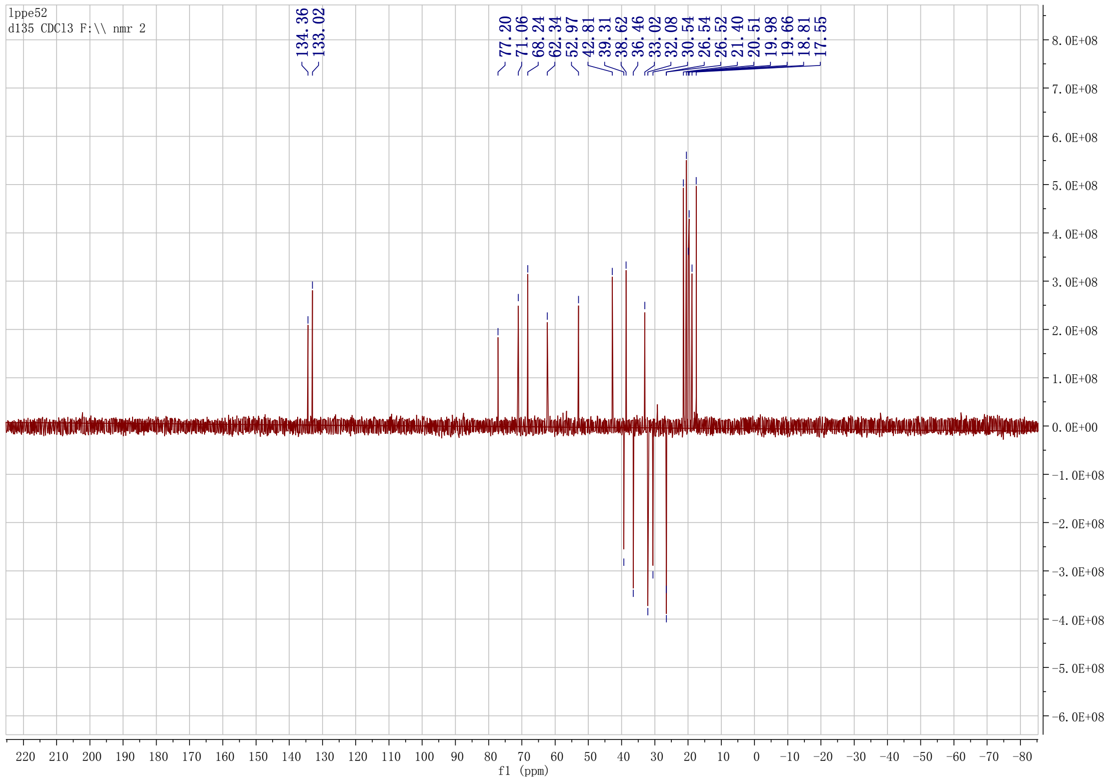
lppe52
d135 CDCl3 F:\\ nmr 2
134.36
133.02
77.20
71.06
68.24
62.34
52.97
42.81
39.31
38.62
36.46
33.02
32.08
30.54
26.54
26.52
21.40
20.51
19.98
19.66
18.81
17.55
f1 (ppm)

HSQC of **4**.

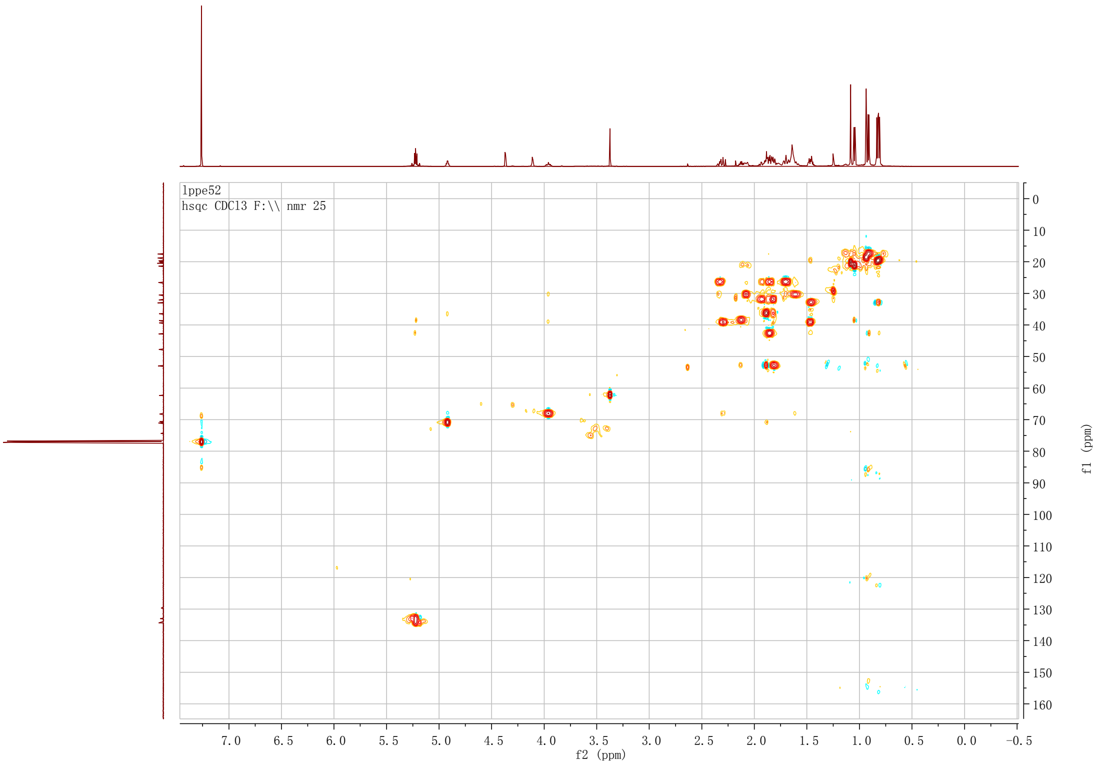

HMBC of **4**.

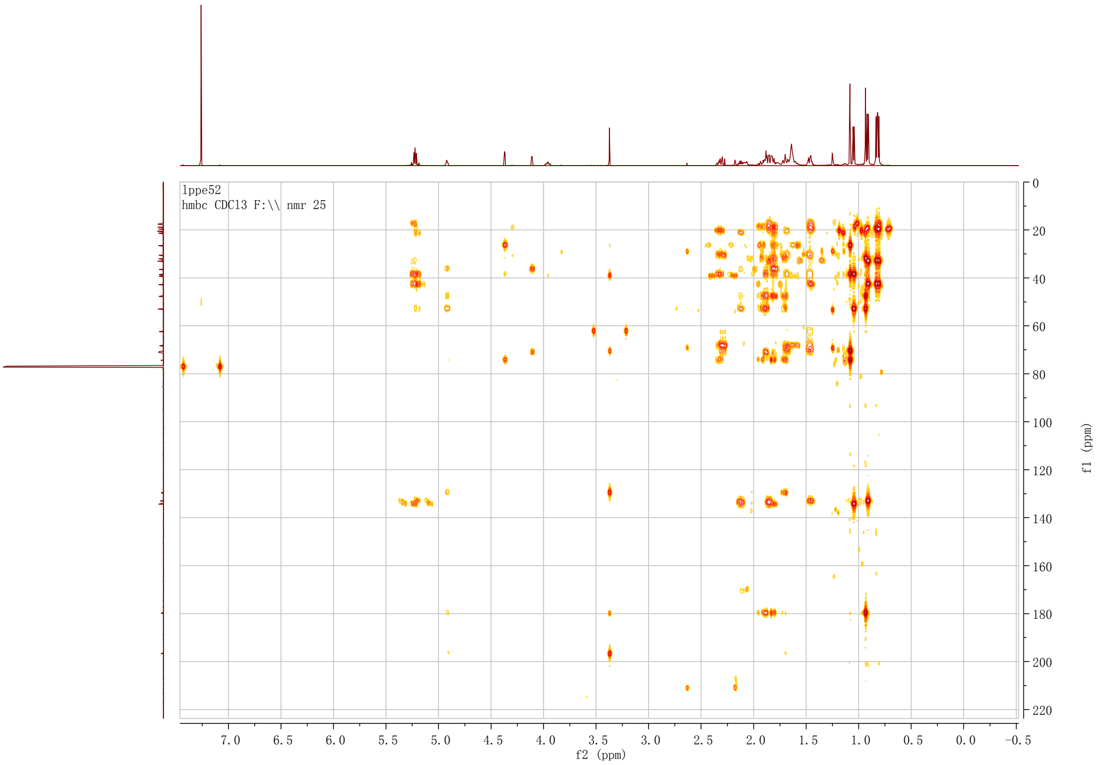

ROESY of **4**.

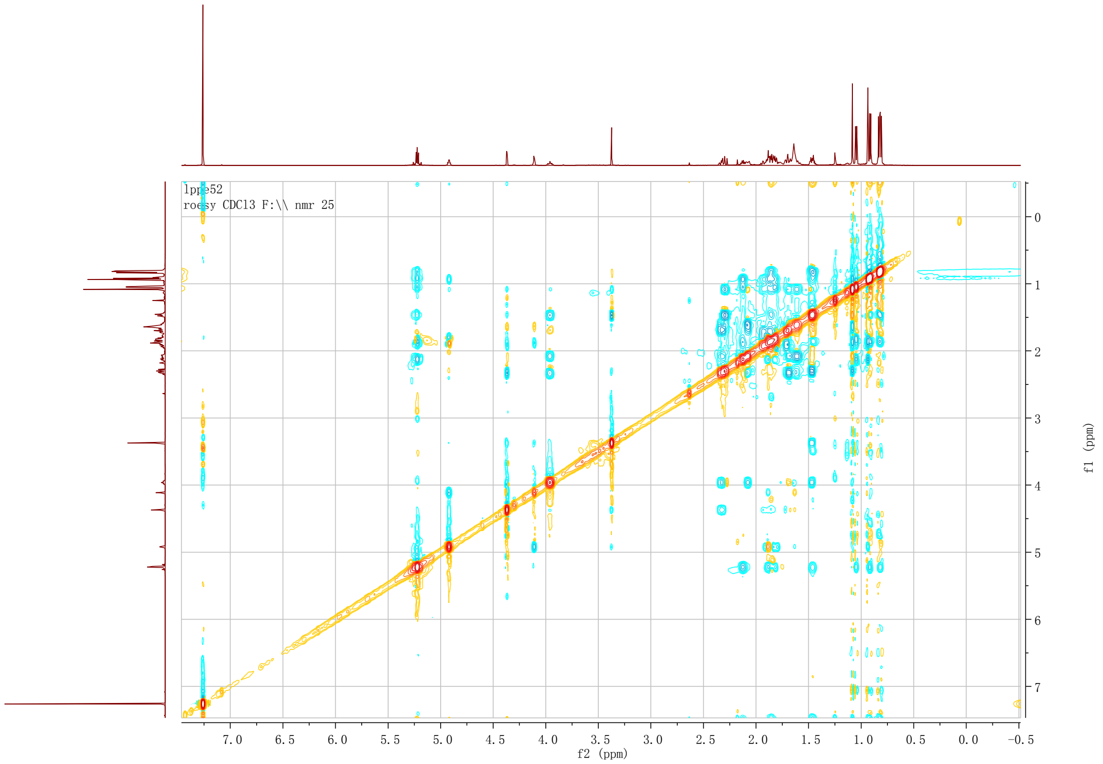

COSY of **4**.

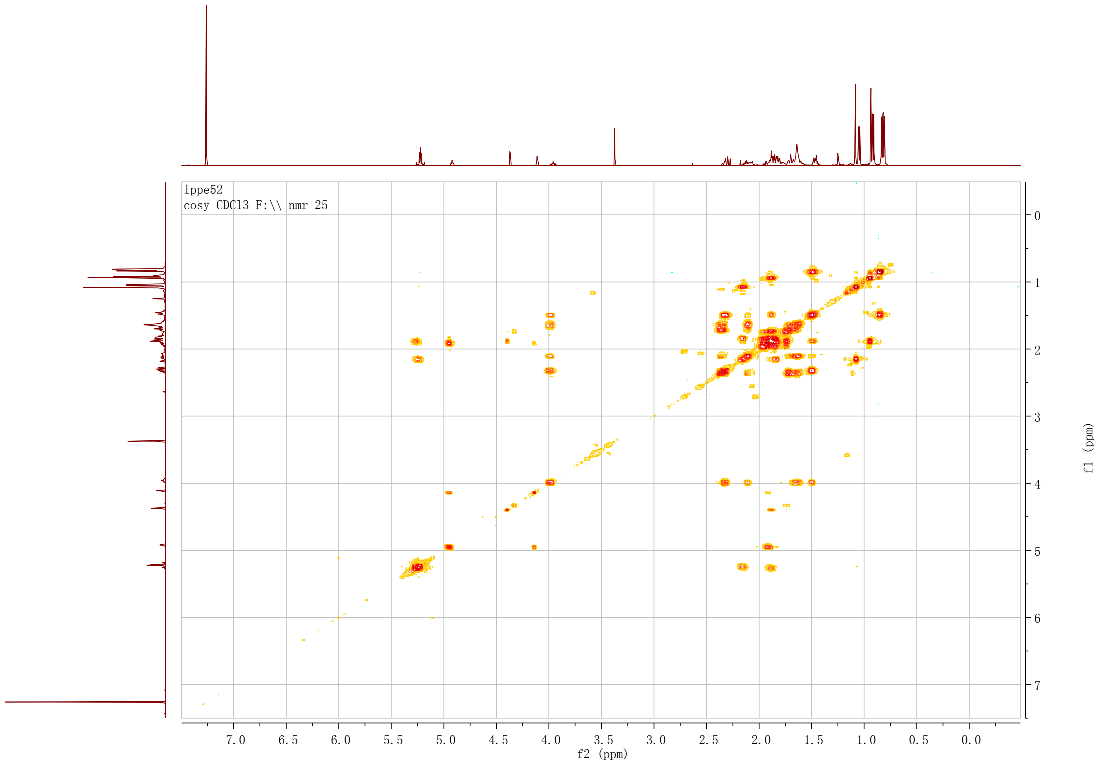

HRESIMS spectrum of **4**.

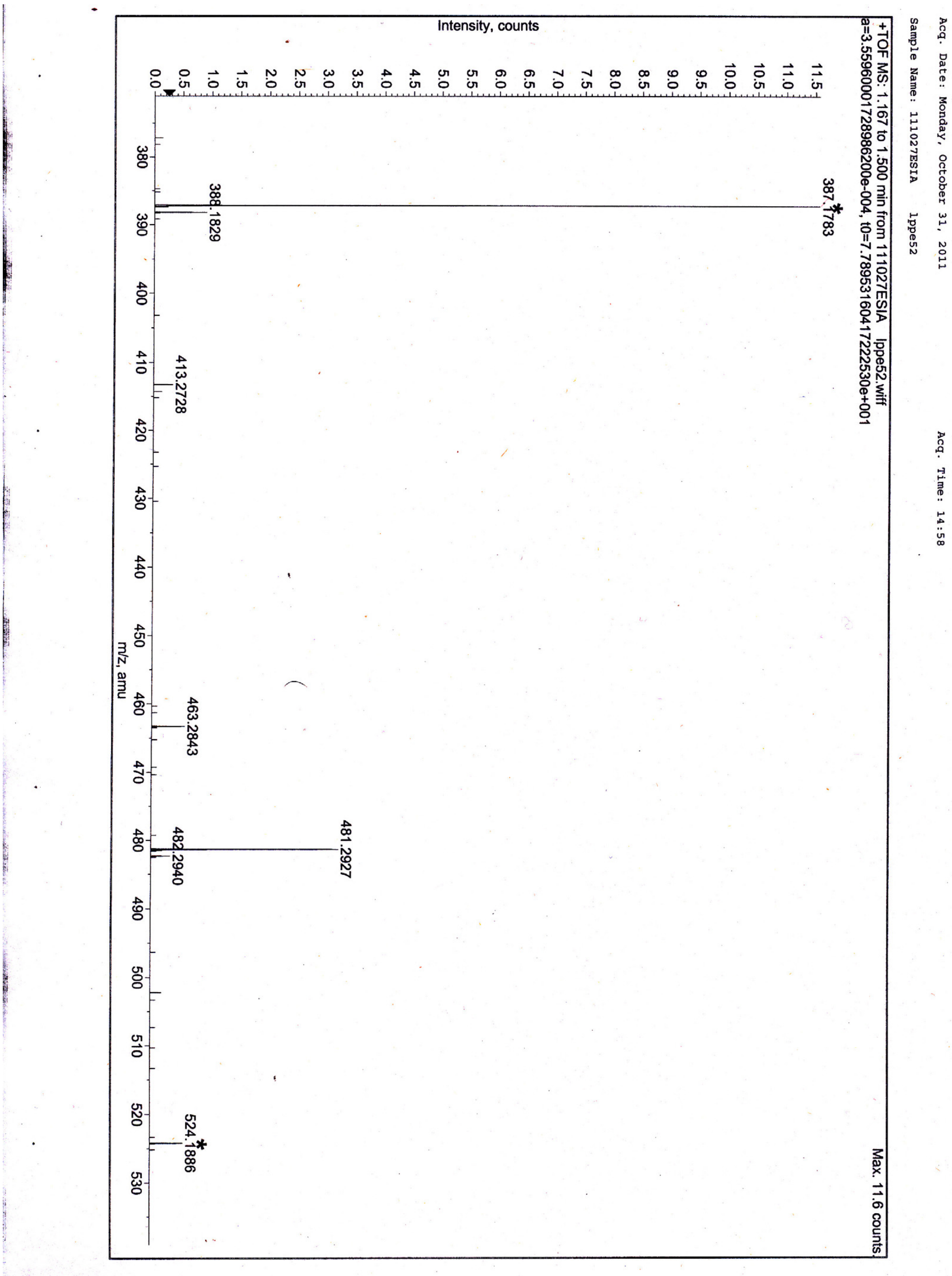

^{1}H NMR spectrum of **5** in $CDCl_3$.

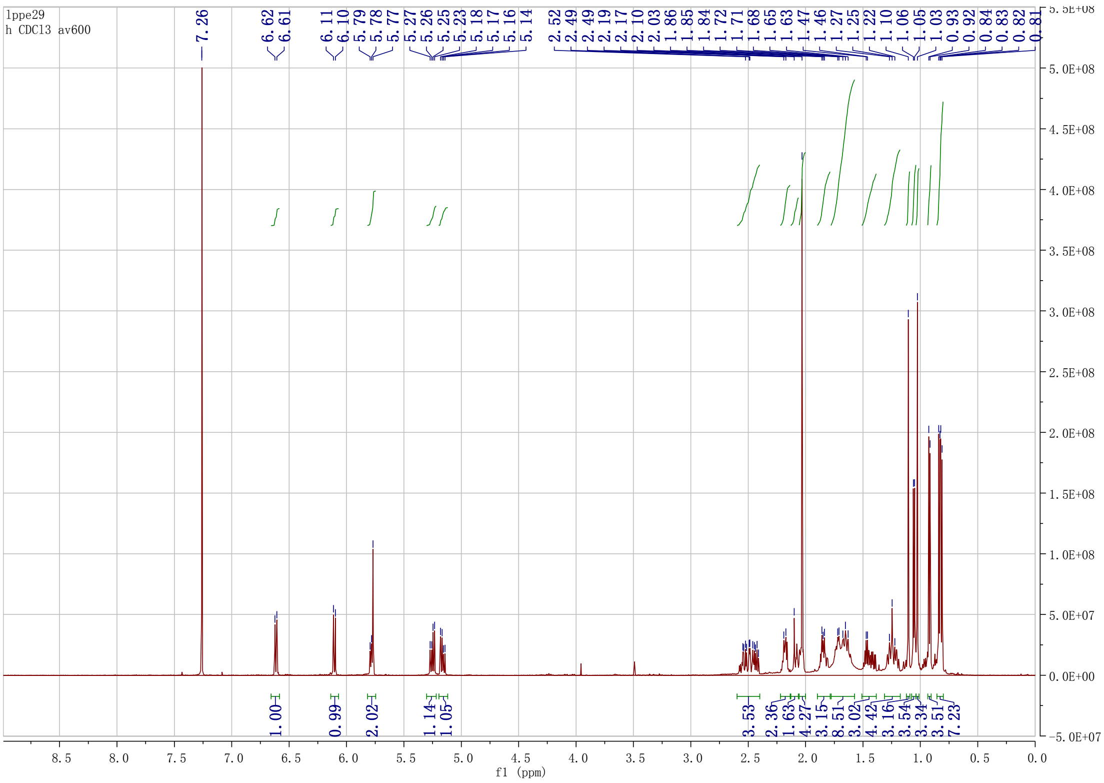

^{13}C NMR and DEPT spectra of **5** in $CDCl_3$.

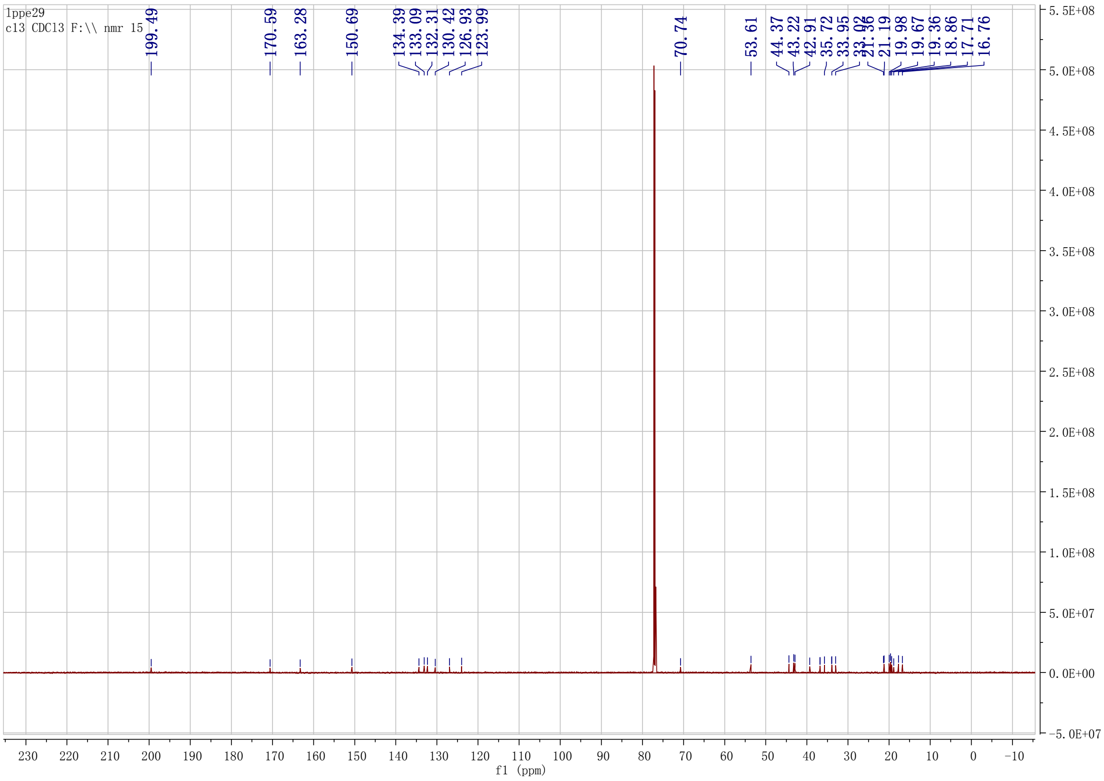

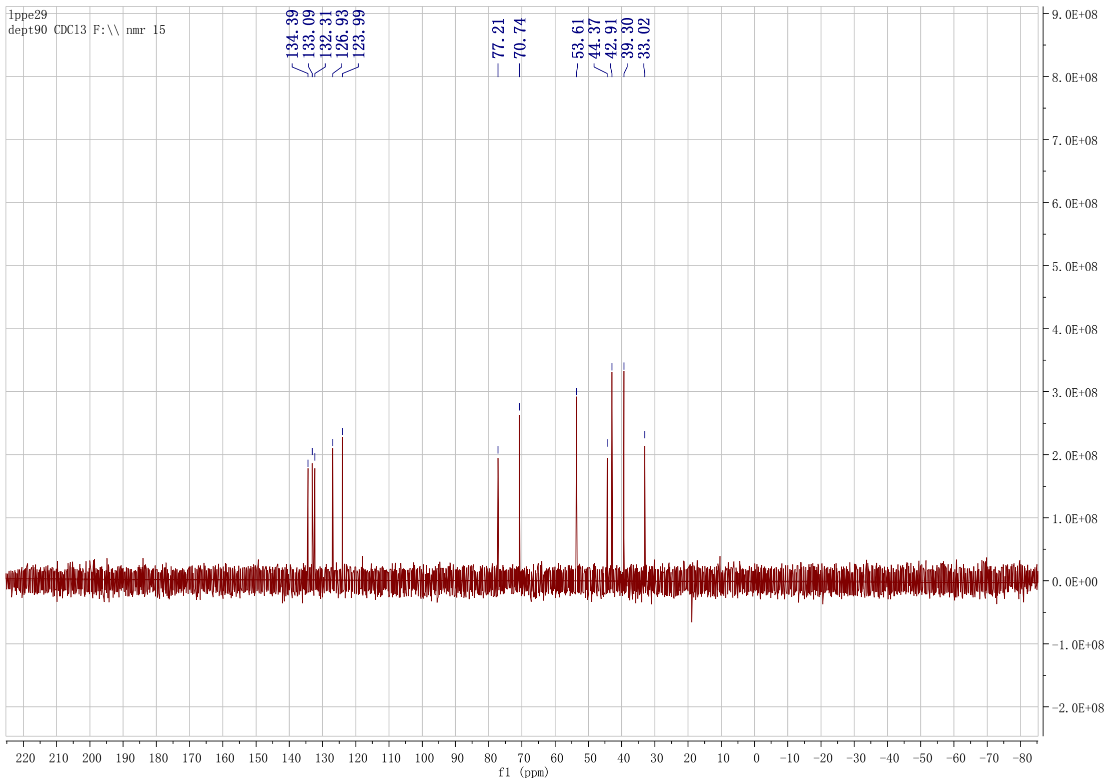
lppe29
dept90 CDCl3 F:\\ nmr 15
134.39
133.09
132.31
126.93
123.99
77.21
70.74
53.61
44.37
42.91
39.30
33.02
f1 (ppm)

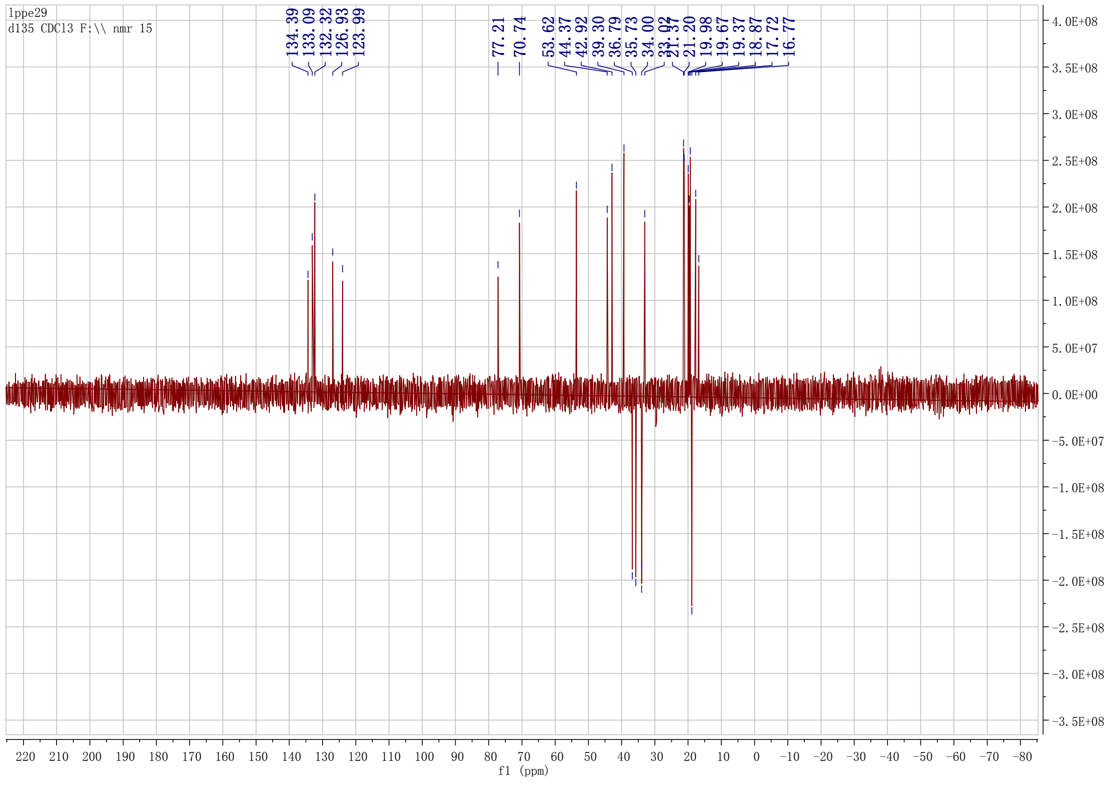
lppe29
d135 CDCl3 F:\\ nmr 15
134.39
133.09
132.32
126.93
123.99
77.21
70.74
53.62
44.37
42.92
39.30
36.79
35.73
34.00
33.02
21.37
21.20
19.98
19.67
19.37
18.87
17.72
16.77
f1 (ppm)

HSQC of **5**.

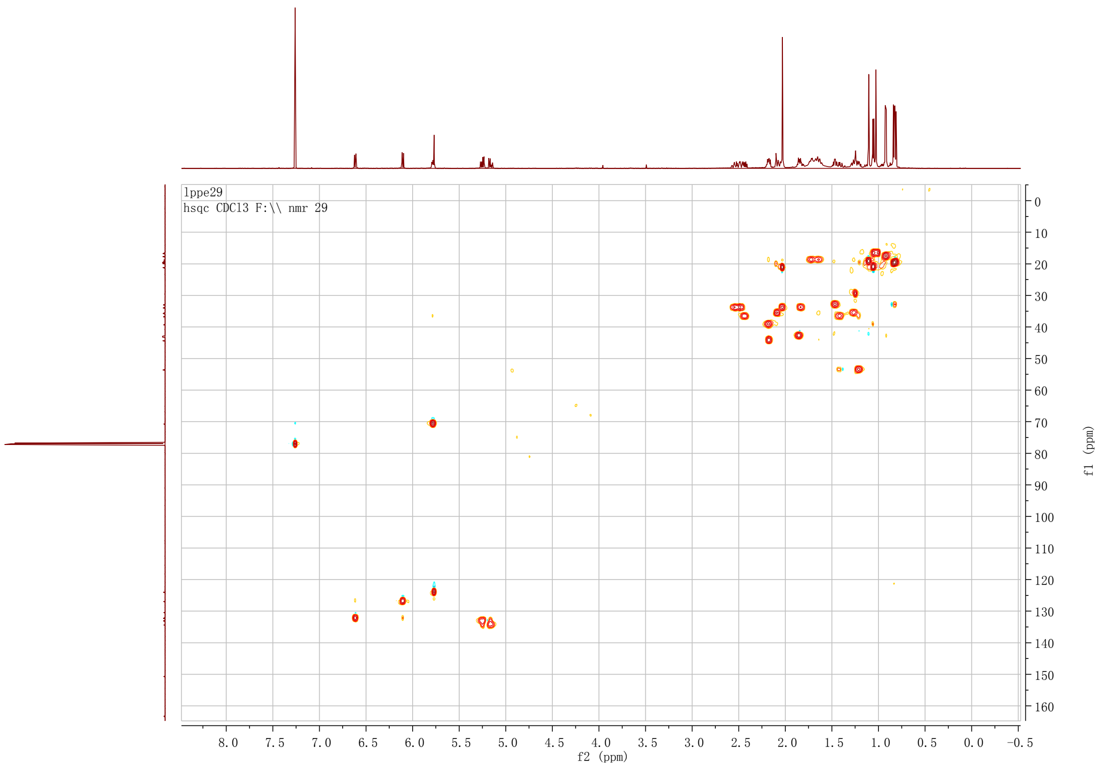

HMBC of **5**.

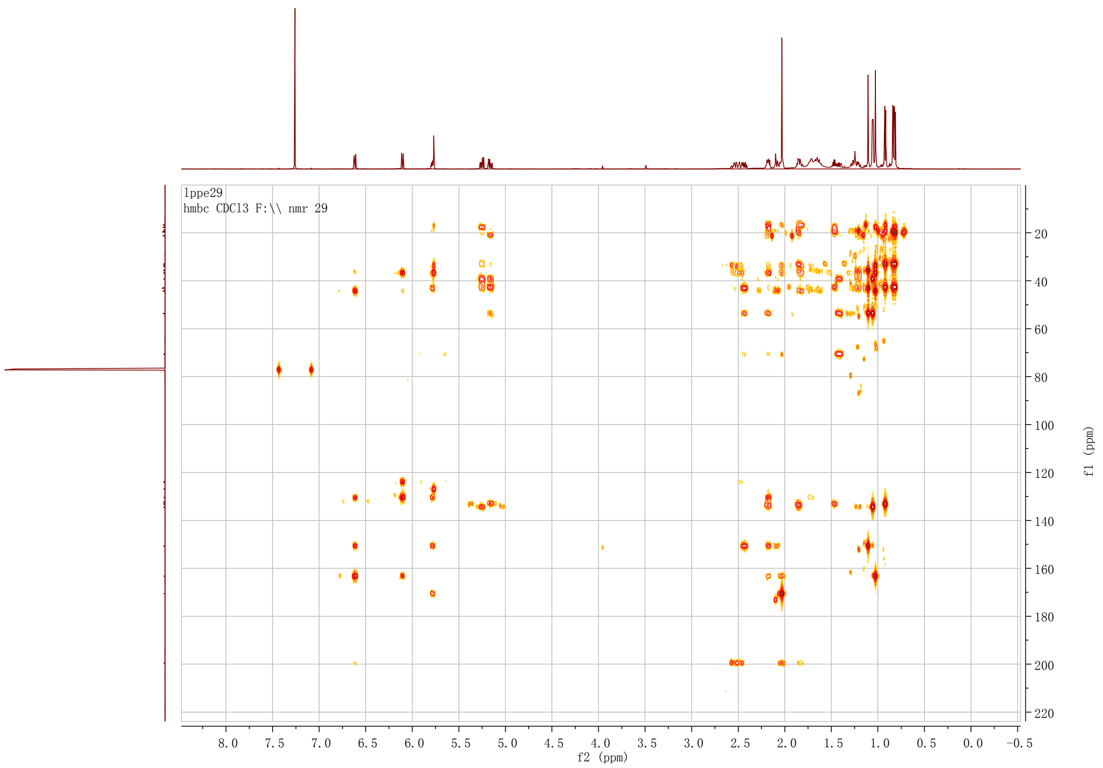

ROESY of **5**.

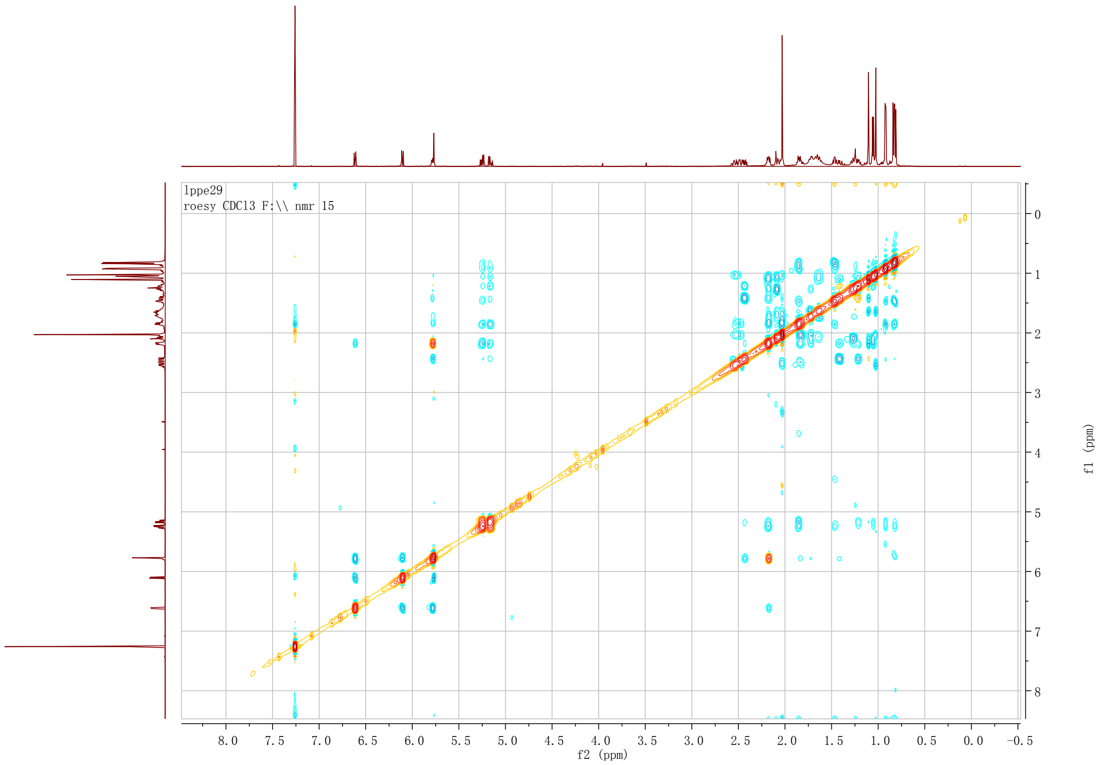

HRESIMS spectrum of **5**.

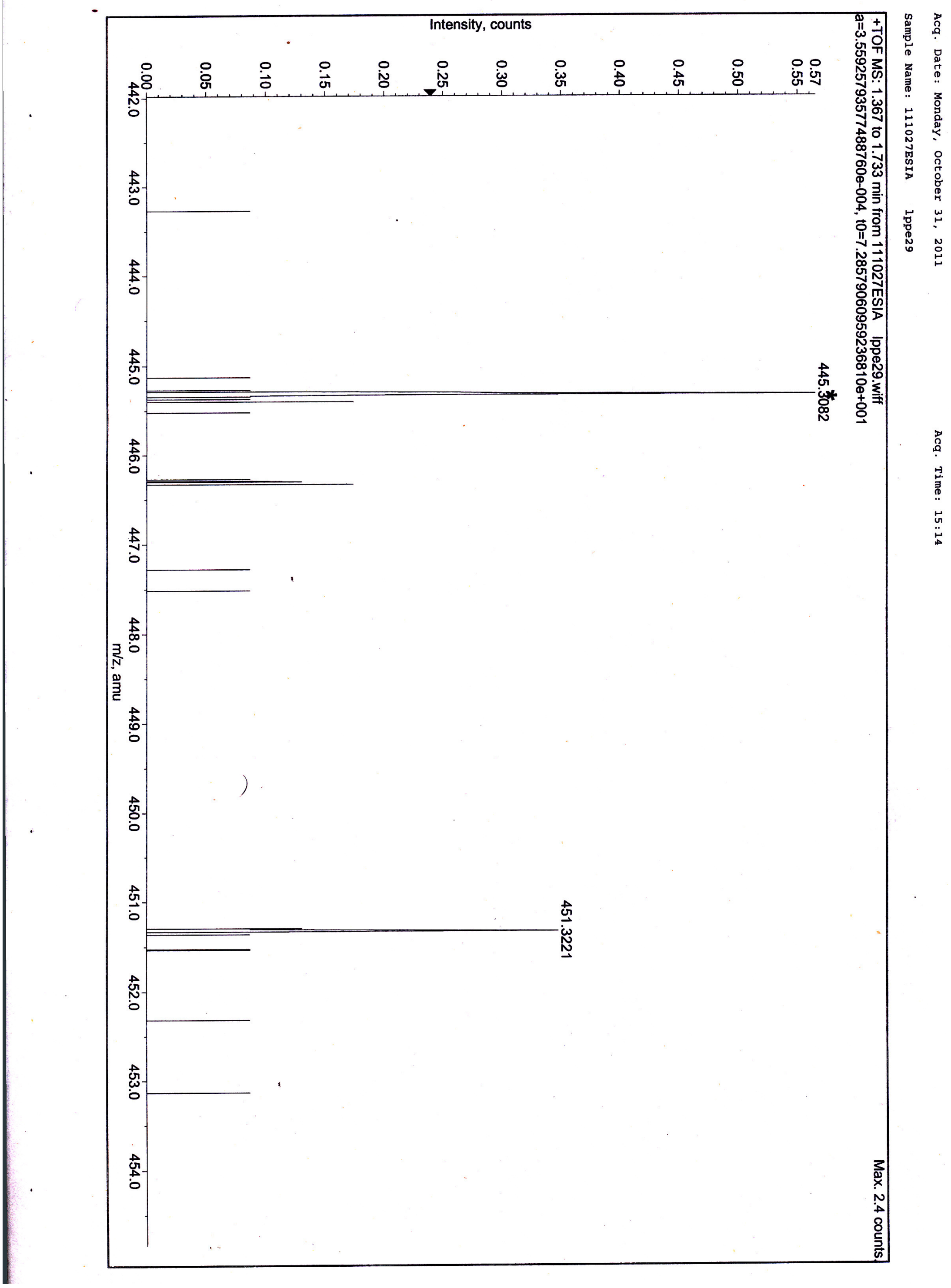

^{1}H NMR spectrum of **6** in $CDCl_3$.

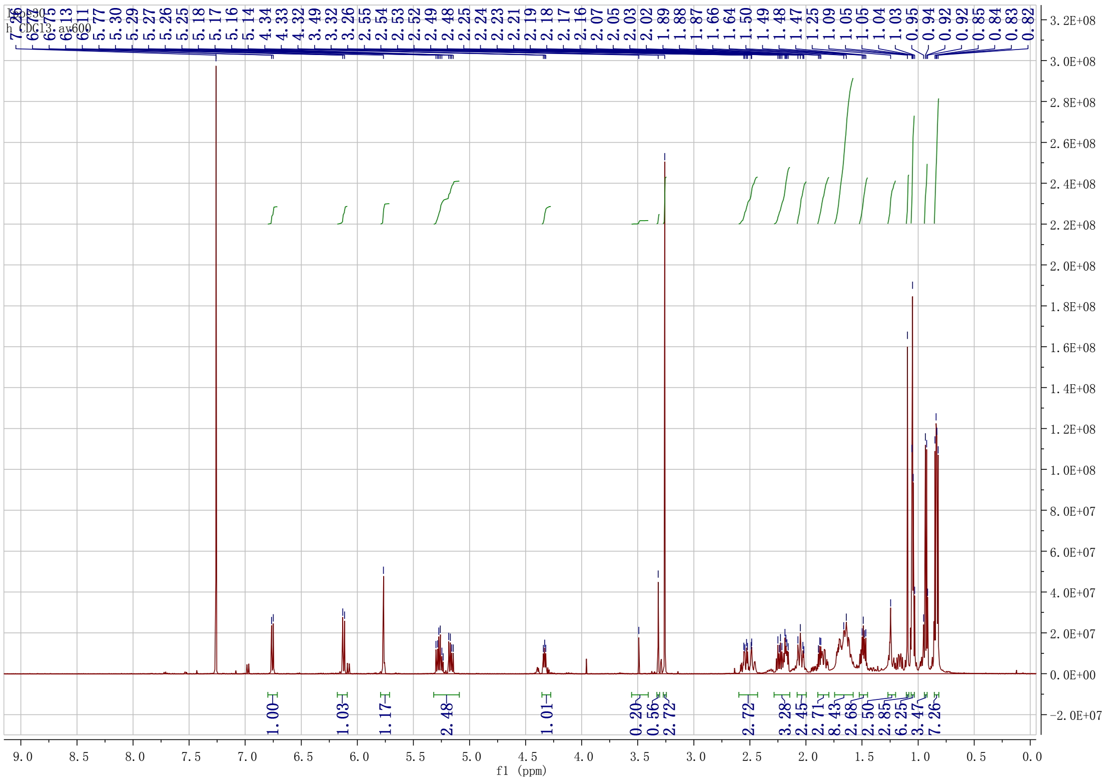

^{13}C NMR and DEPT spectra of **6** in $CDCl_3$.

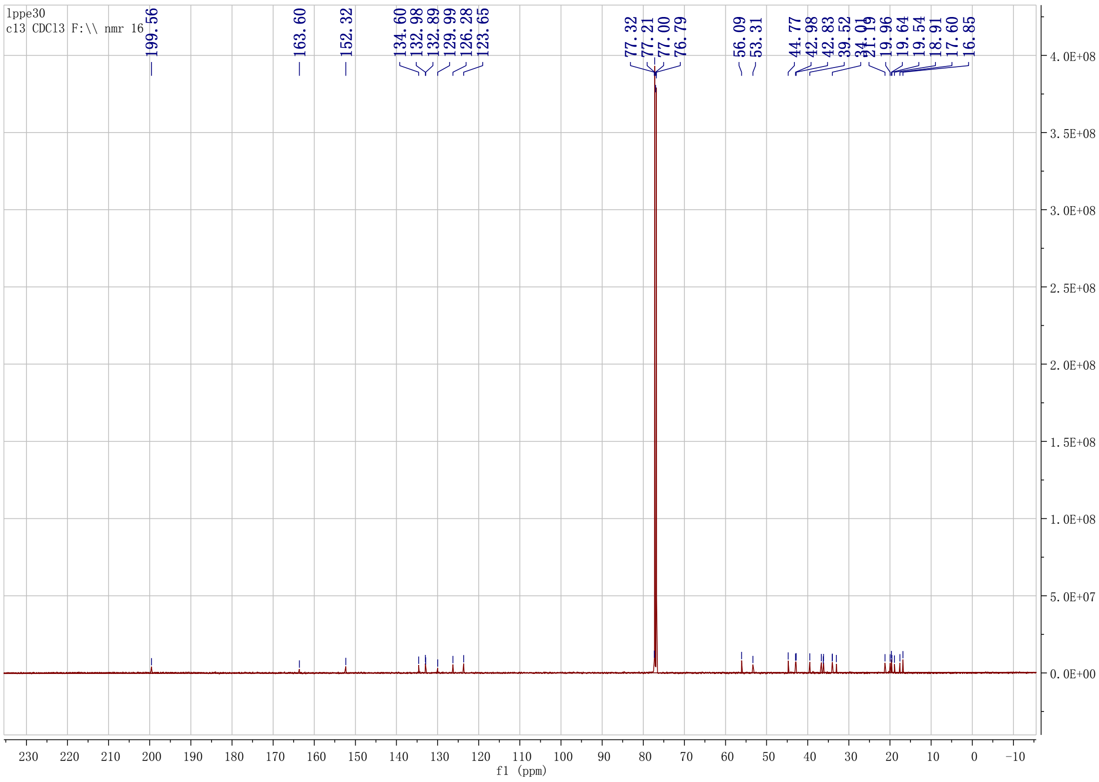

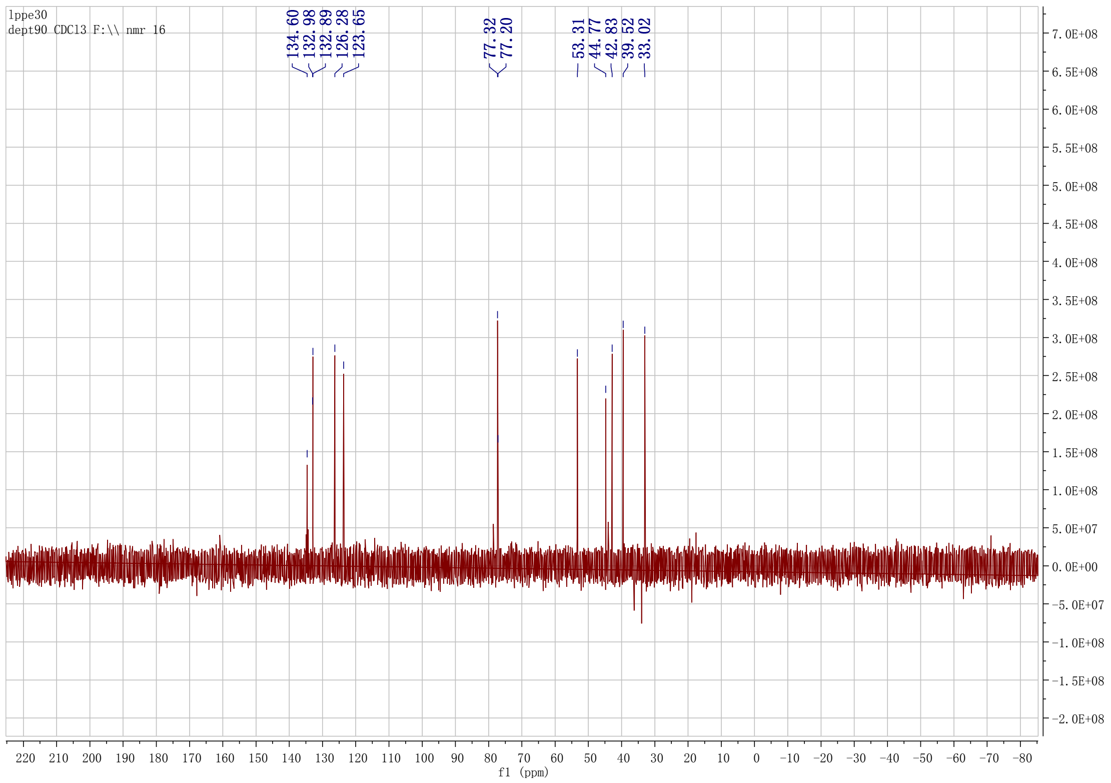
lppe30
dept90 CDCl3 F:\\ nmr 16
134.60
132.98
132.89
126.28
123.65
77.32
77.20
53.31
44.77
42.83
39.52
33.02
f1 (ppm)

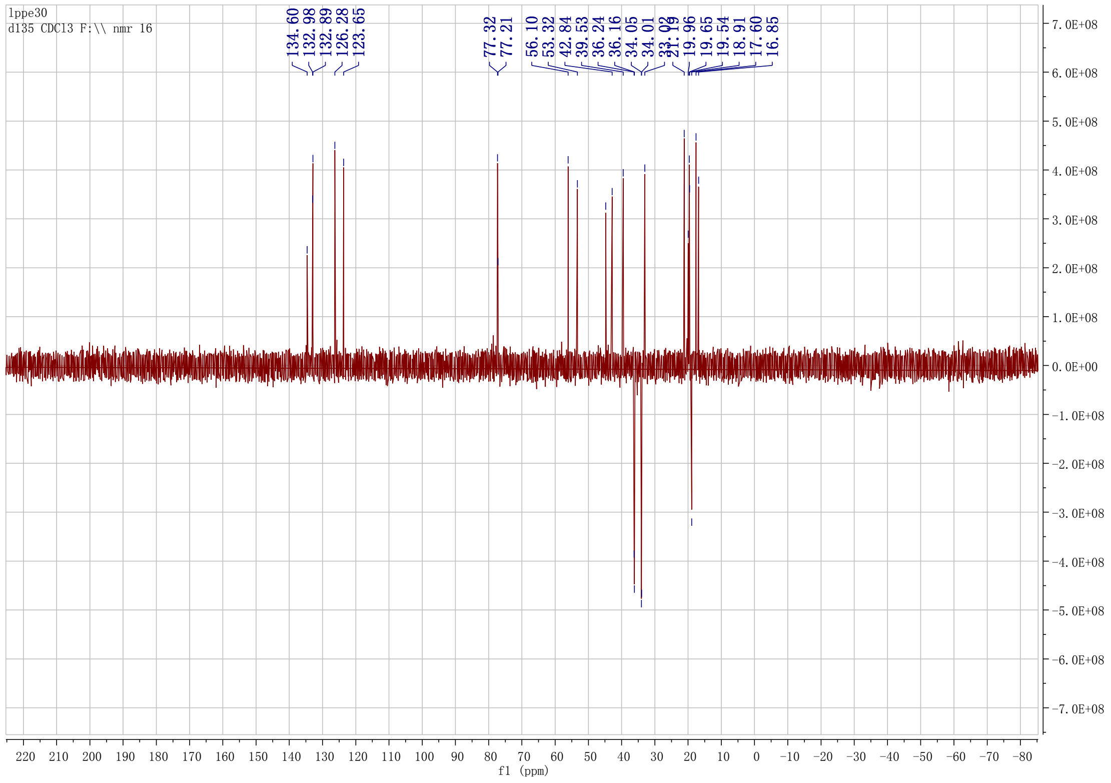
lppe30
d135 CDCl3 F:\\ nmr 16
134.60
132.98
132.89
126.28
123.65
77.32
77.21
56.10
53.32
42.84
39.53
36.24
36.16
34.05
34.01
19.96
19.65
19.54
18.91
17.60
16.85
f1 (ppm)

HSQC of **6**.

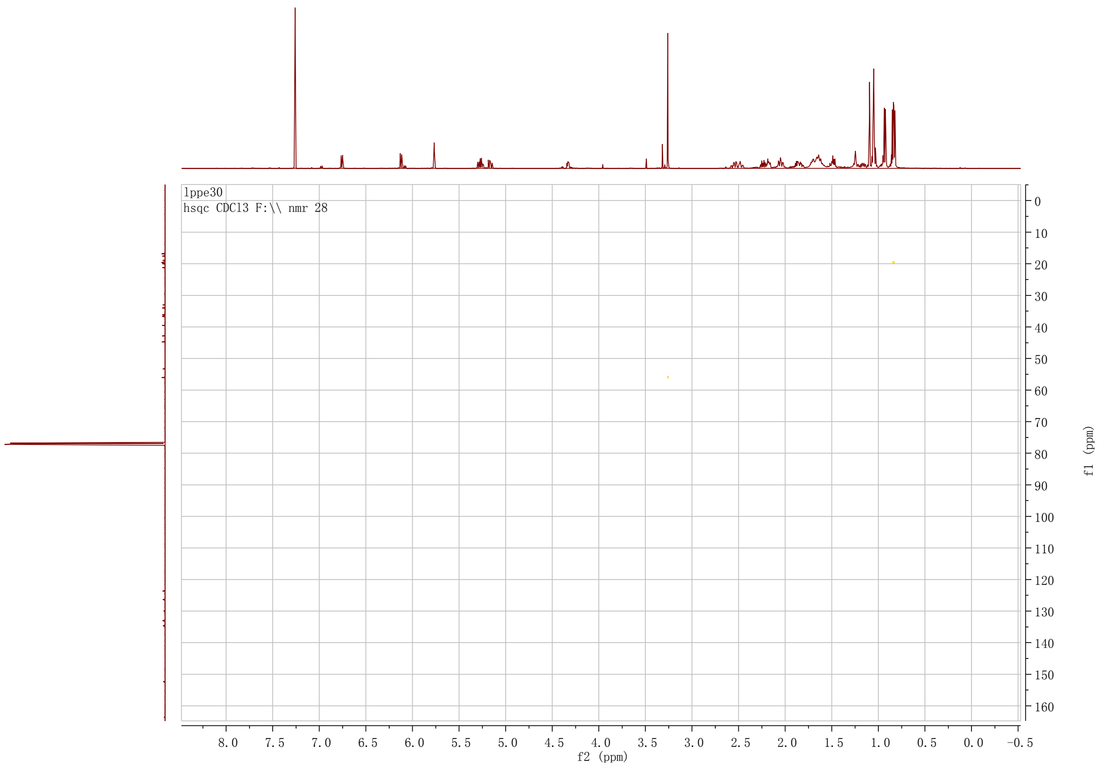

HMBC of **6**.

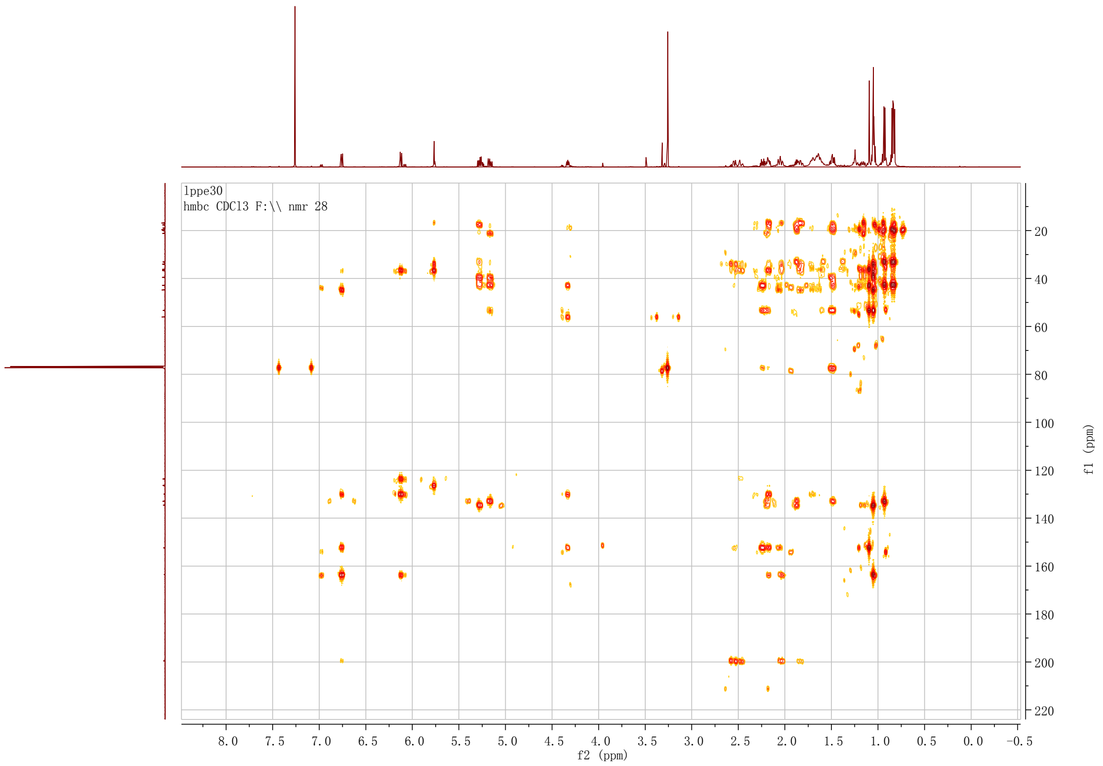

ROESY of **6**.

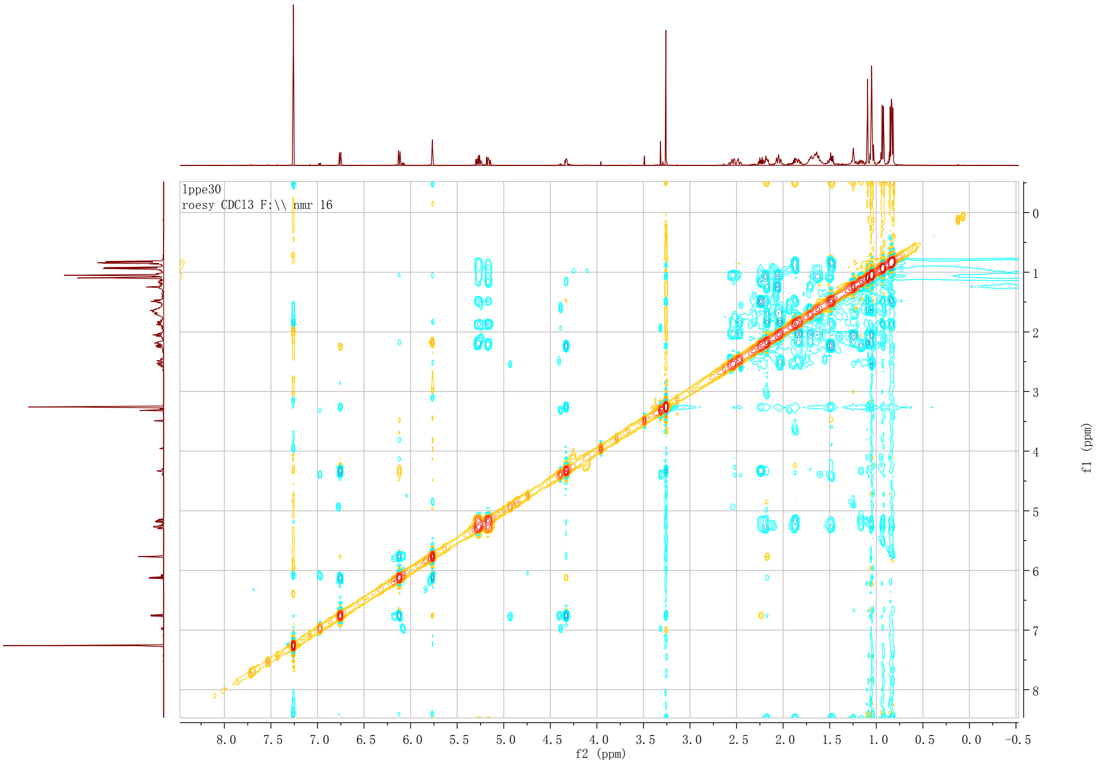

1ppe30
roesy CDCl3 F:\\ nmr 16
8.0 7.5 7.0 6.5 6.0 5.5 5.0 4.5 4.0 3.5 3.0 2.5 2.0 1.5 1.0 0.5 0.0 -0.5
f2 (ppm)
0 1 2 3 4 5 6 7 8
f1 (ppm)

HRESIMS spectrum of **6**.

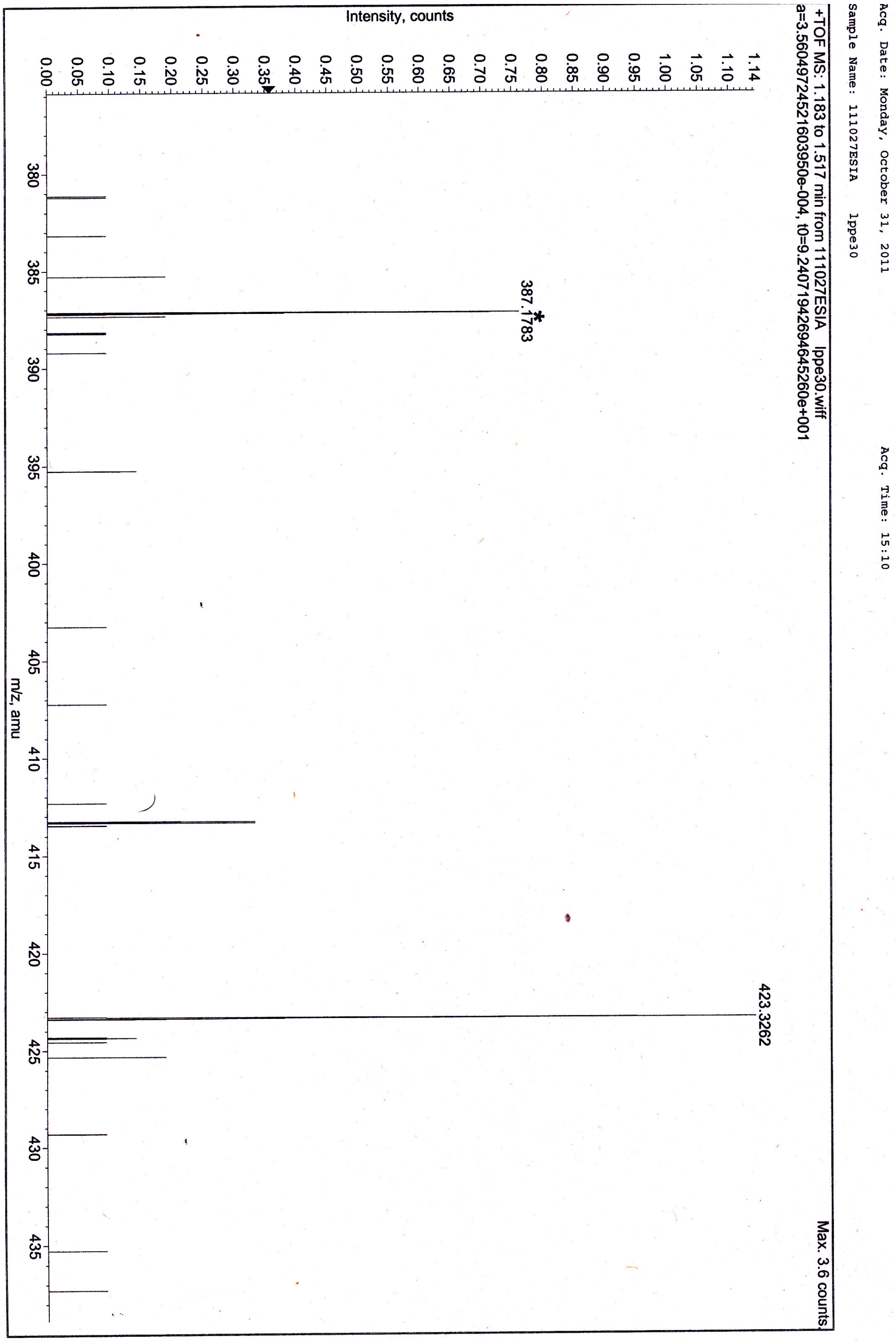

Supplement: Supplementary file 1 — Supplementary material, approximately 6.54 MB. [file 13659_2012_58_MOESM1_ESM.pdf]
